# Validation of the immersion in digital life and quality of digital experience scales in German, French, Spanish, Polish and Czech

**DOI:** 10.3389/fpsyt.2025.1645260

**Published:** 2026-02-19

**Authors:** Joanna Witowska, Christine Schoetensack, Katarzyna Goncikowska, Sébastien Chappuis, Mónica Fernández Boente, Tereza Klegr, Julie Papastamatelou, Rafael Valenzuela, Vanda Černohorská, Nuria Codina, Chantal Martin-Soelch, Marc Wittmann, José V. Pestana, Georgina Giner, Quentin Meteier, Ruth Ogden

**Affiliations:** 1 Institute of Psychology, The Maria Grzegorzewska University, Warsaw, Poland; 2 School of Psychology, Liverpool John Moores University, Liverpool, United Kingdom; 3 Institute of Psychology, Polish Academy of Sciences, Warsaw, Poland; 4 Clinical and Health Psychology Unit, Department of Psychology, University of Fribourg, Fribourg, Switzerland; 5 Institute of Philosophy, Czech Academy of Sciences, Prague, Czechia; 6 Institute for Frontier Areas of Psychology and Mental Health, Freiburg, Germany; 7 Department of Social Psychology and Quantitative Psychology, University of Barcelona, Barcelona, Spain; 8 Serra Húnter Programme, Barcelona, Spain

**Keywords:** cross-cultural validation, immersion, digital experience, digital technology, measurements, scale development

## Abstract

The Quality of Digital Experience Scale (QDES) and the Immersion in Digital Life Scale (IDLS) were developed to measure positive and negative experiences of digital technology use and the extent to which different facets of life were digital. Critically, the QDES and ILDS were developed to be independent of digital device or platform, thus providing a more holistic account of digital technology use than previous measures. The objective of the current studies was to validate the QDES and IDLS in German, French, Czech, Polish and Spanish. Across the studies, data from a total of 4,447 participants were analyzed. Confirmatory Factor Analyses confirmed a three-factor model for the QDES consisting of Wellbeing, Social Connectedness and Time and Efficiency. The IDLS was also confirmed as having a single factor structure. Reliability and validity analysis indicated that the QDES and IDLS showed good reliability and validity in all countries. The present study confirms that the QDES and IDLS offer reliable measurements of individuals’ interactions with technology in the digital era. By extending the validation of these measures beyond English, to German, Polish, Czech, French and Spanish, we expand researchers and policy makers abilities to establish the positive and negative impacts of digital practices on individuals and societies.

## 1 Introduction

One of the key issues facing society today is how we understand and manage the impact of digital technology (DT) on health and wellbeing (1, 2). One difficulty academics face when trying to establish precisely how DT impacts health and wellbeing is how we measure the myriad of ways in which people use DT in their day-to-day lives (3). DT is no longer confined to our workplaces; it is embedded into the fabric of our homes, social lives, education, physical health, and leisure (4, 5). DT is also no longer tethered to a particular physical location; the rapid adoption of smartphones, watches and cars means that, for many people, wherever they are and whatever they are doing, there is the potential for DT to feature. Establishing the impact of a factor which is present to a greater or lesser degree in almost all aspects of life requires a measurement tool which not only examines the extent to which DT is used, but also the experience of its use.

To date, existing measures of DT have limited the extent to which multiple, often overlapping forms of DT are used and embedded into everyday life (6). This is because they have focused on measuring the use of specific technologies (7–9), such as platforms or apps (10). As a result, these measures are only able to capture the impact of one specific form of DT use, typically in one specific area of life (e.g. work or home) (11–13) rather than multiple forms in multiple environments. Critically, because these measures are device or platform specific, they often become rapidly outdated because the speed at which new digital innovations are created greatly outweighs the capacity of researchers to develop validated measures.

Current measures of DT use also have a tendency to focus on problematic or addictive forms of behavior, rather than everyday user experience which is comparably non-problematic (9). As a result, the data collected often highlights the negative experiences of specific populations rather than the impact of digital experience as a whole. They also often rely on estimates of the duration of use (14), rather than exploring the broader experience of use, thus giving limited insight into the impact of DT on broader psychological function.

To overcome these issues, Witowska et al. (6) recently developed and validated two new measures of DT use and experience: the Immersion in Digital Life Scale (IDLS) and the Quality of Digital Experience Scale (QDES). The IDLS is a 5-item scale which measures the extent to which different elements of life are completed digitally (e.g. communications, free time, social life). Immersion in digital life refers to the extent to which individuals engage in the online world. Immersion is a characteristic of an individuals’ behaviour that may depend on personality trait (e.g. 75), differing needs, goals, and beliefs (73) and age-independent levels of digital literacy and digital fluidity (74). The scale captures subjective judgements of the extent to which different activities of everyday life are carried out digitally and does not focus on the frequency or duration of DT use, e.g. minutes or hours of use or another objective indicator.

The QDES is a 26-item tool which measures the quality of day-to-day DT use through three subscales: Wellbeing, Social Connectedness and Time and Efficiency. Quality of digital experiences is understood as individuals’ perceptions and overall experience of their DT usage and its positive impact on core aspects of life. The Wellbeing subscale refers to the degree to which digital technology contributes to mental health, positive mood, relaxation, and overall enjoyment of life. This construct captures the beneficial influence of digital technologies on psychological functioning. The Social Connectedness subscale describes the extent to which digital technology strengthens and supports social ties, promoting a sense of belonging, inclusion, and community. This construct reflects the positive role of digital technologies in enhancing social relationships. The Time and Efficiency subscale measures the degree to which digital technology improves efficiency and supports the completion of daily tasks. This construct highlights the positive impact of digital technologies on the speed, flexibility, convenience, and effectiveness of everyday activities (6).

Critically, the IDLS and QDES overcome the issues associated with previous measures of DT use because they are 1) device and situation independent, enabling the broad impact of multiple forms of ever developing DT to be measured, 2) explore digital experiences neutrally and are not specific to pathological users, and 3) do not rely solely on retrospective estimates of usage time, instead focusing on the quality of experience and extent of immersion.

Witowska et al. (6) developed the items for IDLS and QDES from thematic analysis of data from semi-structured interviews exploring day-to-day DT use (study 1). These interviews were conducted with 300 people from the UK, Spain, Germany, Czechia, Poland and Switzerland. The suitability of these items was then assessed using confirmatory factor analysis (CFA), as well as measures of convergent and divergent validity (study 2). The factors and their validity were then reconfirmed in a further study conducted on a sample representative of the UK in terms of age and gender. These studies all showed that the IDLS and QDES have a reliable factor structure and good internal and external validity.

However, the IDLS and QDES have so far only been validated in one cultural context just like many other measures of non-problematic DT use. For example, instruments assessing social media engagement have been validated for use in Germany (e.g. the Social Media Activity Questionnaire by 57), Spain (e.g. the Spanish Facebook Battery by 58) and Poland (see the Social Media Escapism Scale in 59). By contrast, psychometrically robust scales measuring any non-problematic forms of digital engagement seem to be extremely scarce in Czechia and Switzerland, where studies investigating everyday DT use almost always rely on methods of DT use assessment that have not been psychometrically tested (e.g. 60–63). Currently, cross-culturally valid measures of DT use are available for the assessment of problematic digital engagement (e.g. see 64–66), yet measures focusing on ordinary forms of DT use and experience that are suitable for use in different countries are lacking.

The current paper presents five studies which validate the IDLS and QDES in five further populations and languages (German, French, Spanish, Polish and Czech). Each study involved a cross-sectional online survey that assessed Immersion in Digital Life (IDL) and Quality of Digital Experience (QDE) using translated versions of the IDLS and QDES, and measured problematic internet use, emotional wellbeing, satisfaction with life and impulsivity in members of the general population of Germany, French-speaking Switzerland, Spain, Poland or Czechia. CFAs were performed for all five language versions of the IDLS and QDES, followed by an assessment of their reliability and convergent and discriminant validity. While emotional wellbeing and satisfaction with life were assessed by means of the same instruments in all five studies, problematic internet use and impulsivity were measured using different scales across studies due to the absence of a single validated measure in all five languages.

In line with Witowska et al. (6), we decided to assess the above-mentioned four variables (emotional wellbeing, satisfaction with life, problematic internet use and impulsivity) to explore their associations with IDL and QDE. The variables hold importance in cyberpsychology research (68) and were also assessed in the original validation study of the QDES and IDLS (6). Emotional well-being is understood as positive feelings about current mental state and satisfaction with life is defined as a global perception of life and refers to a cognitive judgement of one’s relation. These constructs were included in the current study as they are indicators of general wellbeing and are therefore likely to be associated with digital wellbeing as well as positive digital experiences assessed by the QDES. Problematic internet use is one of the most frequently measured constructs in quantitative studies of digital technology use that is understood as an excessive and uncontrolled usage causing psychological issues or a type of addiction (67). Despite focusing on “abnormal” use of DT rather than everyday use, it was considered to be sufficiently closely related to the constructs measured by the IDLS and QDES. We also decided to measure impulsivity as it was expected to be a factor correlated with general immersion in digital life (6).

In line with the findings of Witowska et al. (6), it was hypothesized that IDL would correlate positively with global QDE and QDE Wellbeing, Social Connectedness and Time and efficiency. Furthermore, global QDE was expected to show positive associations with each of its three subdimensions. In addition, positive relationships of IDL and QDE with problematic internet use were anticipated.

Considering the correlations of IDL and QDE with indicators of both positive and negative wellbeing reported in Witowska et al. (6) and the absence of a correlation with some wellbeing variables, it was assumed that the relationships of IDL and QDE with emotional wellbeing, satisfaction with life and impulsivity might be similar but not identical to those reported in Witowska et al.’s (6) study.

## 2 Study 1: validation of the IDLS and QDES in German

### 2.1 Materials and methods

#### 2.1.1 Participants

A sample of 1226 individuals representative in terms of age and gender of the population of Germany participated in the study. 397 participants were excluded for incorrectly answering one or more of three attention check questions included in the questionnaire. The analyses presented are based on a final sample of 829 participants after data exclusions. See **Table 1** for sample details.

**Table 1:** Sample details for the 5 studies reported.

Variables	1 German	2 French	3 Spanish	4 Polish	5 Czech
Initial sample	1226	878	1224	1208	1216
Participants removed for failing attention checks	397	231	429	0	248
Final sample	829	647	795	1208	968
Mean age (SD)	47.59 (13.14)	45.29 (14.96)	46.43 (12.77)	42.28 (12.94)	45.23 (13.33)
Gender					
Male	414	307	381	604	445
Female	412	337	412	603	520
Other gender identity or no gender indicated	3	3	2	1	3
Highest level of education					
Below degree level	453	159	213	566	321
Technical qualifications	415	175	237	229	147
University degree	346	239	345	413	500
Professional degree or equivalent	0	61	0	0	0
Other	8	10	0	0	0
Prefer not to say	0	3	0	0	0

#### 2.1.2 Procedure

Ethical approval was obtained from Liverpool John Moores University Research Ethics Committee (Approval reference number: 23/PSY/061). Data collection followed the principles of the Declaration of Helsinki and was initiated on 16^th^ October 2023 and completed on 27^th^ October 2023. Recruitment of participants was undertaken by the recruitment platform Qualtrics Panels, which distributed an online questionnaire containing the measures described below to eligible volunteers who had previously indicated a willingness to participate in research. Participants were rewarded for their participation. All participants gave informed electronic consent. Mean study completion time was 14.26 minutes.

##### 2.1.2.1 Translation of the IDLS and QDES

The IDLS and QDES (6) were translated to German using the translation method outlined by Beaton et al. (15). More specifically, the translation involved the following six steps: (1) The generation of forward translations (i.e. from English to German) by two bilingual/professional translators who worked independently, (2) The collaborative production of one optimized forward translation of each measure after thorough discussion and review of the aforementioned translations by the translators and the German research team and resolution of any discrepancies between translations, (3) Back translations (from German to English) of the optimized forward translations by two additional independent bilingual/professional translators, (4) Review and comparison of the original English measures with all produced translations among the research team and agreement on prefinal versions of the IDLS and QDES that showed semantic and idiomatic equivalence with the original English instruments, (5) Collection of feedback on the prefinal versions from three laypeople whose first language was German. The lay panel was instructed to read all items of the measures, identify any items perceived as unclear as well as specific aspects of unclear items in addition to reasons for any lack of clarity. (6) Review and comparison of feedback, adjustment of the prefinal versions of the measures in line with lay feedback and production of the final German translations of the IDLS and the QDES. These are provided in the **Supplementary Material**.

#### 2.1.3 Measures

##### 2.1.3.1 Demographic details

Participants indicated their gender, age and highest level of education. Gender was measured using a multiple-choice question (“what gender do you identify with?”), which presented participants with the five response options “male”, “female”, “divers” (German word meaning “any other gender identity than male or female”), “other (please describe)” and “prefer not to say”.

##### 2.1.3.2 Immersion in digital life

The 5-item Immersion in Digital Life Scale (IDLS) (6) measured immersion in digital life i.e. the extent to which DTs were used in different life domains. The instrument required participants to state how “digital” their social relationships (item 1), communication with family (item 2), free time activities (item 3), communication with friends (item 4), and time management (item 5) were. A visual analogue scale with the two anchors “not at all digital” (left anchor), which corresponded to a score of 0, and “completely digital” (right anchor) associated with a score of 100 was displayed below each item. Participants were instructed to respond to each item by dragging a slider to the desired point on the scale. The questionnaire was scored by calculating the mean of all individual item scores, with higher values suggesting greater immersion in digital life.

##### 2.1.3.3 Quality of digital experience

The 26-item Quality of Digital Experience Scale (QDES) (6) assessed overall lived experience of DT usage and its impact on core aspects of life. The measure is composed of the three subscales Wellbeing (5 items), Social Connectedness (12 items), and Time and Efficiency (9 items). Each item of the questionnaire is phrased as a statement and participants were required to indicate their level of agreement with each by selecting a response from a 5-point Likert scale with the response options 1 (*strongly disagree*), 2 (*disagree*), 3 (*neither agree or disagree*), 4 (*agree*) and 5 (*strongly agree*). The final scores were obtained by calculating the mean of all individual item scores and the average values for each subscale. Greater scores were an indication of a more positive experience of digital engagement.

##### 2.1.3.4 Problematic internet use

The 14-item Compulsive Internet Use Scale (CIUS) (16) measured problematic internet use. Participants indicated how frequently they displayed signs of compulsive internet use on a Likert-scale ranging from 0 (*never*) to 4 (*very frequently*). A total score was obtained by computing sums of individual item scores, with higher scores indicating greater compulsive internet use.

##### 2.1.3.5 Emotional wellbeing

The German version of the WHO-5 Wellbeing Index (WHO-5) (17) was employed as a measure of global emotional wellbeing. The 5-item questionnaire contains statements tapping the perceived amount of time over the past two weeks during which respondents experienced positive mood, relaxation, vitality, good rest and interest in their daily life. Responses were provided on a 6-point Likert scale ranging from 5 (*all the time*) to 0 (*at no time*). The sum of all item scores constituted the total score, with higher numbers implying better emotional wellbeing.

##### 2.1.3.6 Satisfaction with life

The Satisfaction with Life Scale (SWLS) (18) assessed how content participants felt with their lives. The measure prompted respondents to indicate their level of agreement with five statements on a 7-point Likert scale ranging from 7 (*strongly agree*) to 1 (*strongly disagree*). Item scores were summed so that higher total scores indicated greater satisfaction with life.

##### 2.1.3.7 Impulsivity

The 30-item Barratt Impulsiveness Scale 11 (BIS-11) (19) served as a measure of trait impulsivity. The instrument required participants to rate how often they thought or behaved in certain ways by selecting a response from a 4-point Likert scale ranging from 1 (*rarely/never*) to 4 (*almost always/always*). The measure was composed of the subscales Non-planning, Motor and Attentional impulsiveness. Item scores were summed to obtain a total score so that higher scores indicated greater impulsivity. Subscale scores are not reported in our study.

#### 2.1.4 Analytic strategy

All analyses were performed using IBM SPSS AMOS 28 and IBM SPSS 28.

To confirm the structures of the IDLS and QDES reported by Witowska et al. (6), we conducted CFAs (maximum likelihood) of a one-factor model for the IDLS and a three-factor model for the QDES. In accordance with widely used statistical guidelines, we report the following fit indices for each model: the model chi-square (χ2), degrees of freedom (df), CMIN/DF, RMSEA, GFI, CFI and SRMR (20). While building the models we aimed to consider acceptable cut-off points of all the indicators. We prioritized the CFI and SRMR because the RMSEA index is not particularly suitable for models with less than 25 degrees of freedom (21) and the large sample size renders chi-squared liable to type 1 errors (22). Moreover, χ2/df is difficult to interpret because of oversensitivity to sample size (23, 24) and GFI is also affected by sample size (25).

The following criteria for the model fit indicators were used: CMIN/DF <5; RMSEA <.08, GFI> 0.90; CFI >.90 and SRMR <.08 (26–28) although we focus on the CFI and SRMR. We also aimed for item loadings >.40 and close to .70.

Internal consistency of both measures was assessed with Cronbach’s alpha (α), where the threshold of acceptability was .70 (29). The split-half method with even-odd items comparison was additionally conducted. The correlation coefficient (between forms) (r), Spearman-Brown formula (ρ) and Guttmann split-half coefficient (G) were calculated. The higher the value, the better the reliability with an accepted value of at least .70.

Convergent and discriminant validity of the measures were tested by assessing intercorrelations between IDL and QDE as well as correlations of each construct with problematic internet use, emotional wellbeing, satisfaction with life and impulsivity, in accordance with the first validation study of the IDLS and QDES (6).

### 2.2 Results

Descriptive statistics (M and SD) as well as reliability estimates (α) for all constructs measured are reported in **Table 2**.

**Table 2:** Descriptive statistics and reliability for all variables from the German validation.

Variables	Germany		
	*M*	*SD*	*α*
IDL	48.74	22.17	.78
QDE	3.37	0.70	.92
QDE Wellbeing	3.33	0.80	.75
QDE Social Connectedness	3.13	0.88	.89
QDE Time and Efficiency	3.71	0.68	.82
Problematic internet use	20.00	11.73	.93
Emotional wellbeing	10.86	5.86	.90
Satisfaction with life	21.77	7.01	.90
Impulsivity	62.47	10.78	.83

IDL, Immersion in Digital Life; QDE, Quality of Digital Experience; QDE Wellbeing, Quality of Digital Experience (Wellbeing); QDE Social Connectedness, Quality of Digital Experience (Social Connectedness); QDE Time and Efficiency, Quality of Digital Experience (Time and Efficiency).

#### 2.2.1 IDLS

The CFA showed that the χ2 for the model was significant (χ2 = 25.39; df = 5; p <.001). This model shows acceptable indicators of goodness of fit for CMIN/DF and RMSEA (CMIN/DF = 5.07; RMSEA = .07; GFI = .99; CFI = .99; SRMR = .03). Although covariances were added in an attempt to improve model fit, there was no improvement.

**Table 3** shows correlational analyses assessing convergent and discriminant validity. As expected, IDL correlated positively with QDE, QDE Wellbeing, Social connectedness and Time and efficiency. The positive correlation between IDL and problematic internet suggests convergent validity. IDL also correlated positively with impulsivity and satisfaction with life, and negatively with emotional wellbeing.

**Table 3:** Intercorrelations between IDL, QDE and subdimensions of QDE for the German validation.

Germany	IDL	QDE	QDE Wellbeing	QDE Social Connectedness	QDE Time and Efficiency
IDL	–				
QDE	.55**	–			
QDE Wellbeing	.53**	.87**	–		
QDE Social Connectedness	.59**	.93**	.76**	–	
QDE Time and Efficiency	.26**	.79**	.62**	.53**	–
Problematic internet use	.52**	.34**	.32**	.41**	.08*
Emotional wellbeing	-.11**	-.04	-.03	-.06	-.01
Satisfaction with life	.09**	.08*	.07	.09*	.04
Impulsivity	.42**	.15**	.18**	.23**	-.07*

Correlations of IDL, QDE and subdimensions of QDE with relevant measures.

*p <.05; **p <.01.

IDL, Immersion in Digital Life; QDE, Quality of Digital Experience; QDE Wellbeing, Quality of Digital Experience (Wellbeing); QDE Social Connectedness, Quality of Digital Experience (Social Connectedness); QDE Time and Efficiency, Quality of Digital Experience (Time and Efficiency).

#### 2.2.2 QDES

The CFA showed that the χ2 for the model was significant (χ2 = 1061.64; df = 296; p <.001). This model did not show acceptable indicators of goodness of fit for GFI (CMIN/DF = 3.59; RMSEA = .06; GFI = .76; CFI = .95; SRMR = .05). To improve the model fit, we added covariances step by step starting with the highest and checking goodness of fit after every step. The first covariance was added (CMIN/DF = 3.59; RMSEA = .06; GFI = .76; CFI = .95; SRMR = .05). No other covariances could improve the fit indices. While GFI still did not meet the required standard, the rest of the indicators met the requirements for goodness of fit. No items were removed and one residual stayed in the final model. **Figure 1.2** in the **Supplementary Material** presents the final model.

Internal consistency and split-half reliability of the full QDES (α = .92; r =.91; ρ = .95; G = .95) as well as the Wellbeing (α = .75; r = .73; ρ = .84; G = .82), Social Connectedness (α =.89; r = .88; ρ = .94; G = .93) and Time and Efficiency (α =.82; r = .81; ρ = .89; G = .88) subscales were acceptable.

The correlational analysis in **Table 3** shows that QDE correlated positively with QDE Wellbeing, Social connectedness and Time and efficiency. QDE also correlated positively with problematic internet use, suggesting convergent validity. QDE also correlated positively with satisfaction with life and impulsivity. No correlation was observed between emotional wellbeing and QDE.

## 3 Study 2: validation of the IDLS and QDES in French

### 3.1 Materials and methods

#### 3.1.1 Participants

A sample of 878 individuals representative in terms of age and gender of the population of Switzerland participated in the study. 231 participants were excluded for incorrectly answering one or more of three attention check questions included in the questionnaire. The analyses presented are based on a final sample of 647 participants after data exclusions. See **Table 1** for sample details.

#### 3.1.2 Procedure

Data collection was initiated on 16th October 2023 and completed on 31st October 2023. Mean study completion time was 22.06 minutes. The procedure was identical to that of Study 1.

*Translation of the IDLS and QDES*

The IDLS and QDES were translated to French following the same method and procedure as outlined for Study 1. For step 5, feedback on the prefinal versions of the questionnaires was collected from nine laypeople whose first language was French. The final copies of the IDLS and QDES used in this study are provided in the **Supplementary Material**.

#### 3.1.3 Measures

Demographic details, IDL, QDE, emotional wellbeing and satisfaction with life were measured using French versions of the demographic questionnaire, the IDLS, the QDES, the WHO-5 (30) and the SWLS (31), respectively. Please see the section “Measures” of study 1 for a description of these instruments.

##### 3.1.3.1 Demographic details

Gender was measured using a multiple-choice question as in study 1 and six response options (“male”, “female”, “non-binary”, “transgender”, “other (please describe)” and “prefer not to say”).

##### 3.1.3.2 Problematic internet use

Problematic internet use was assessed by the French 9-item Problematic Internet Use Questionnaire 9 (PIUQ-9) (32). Participants indicated the frequency with which they experienced signs of problematic engagement with the internet using a 5-point Likert scale ranging from 1 (*never*) to 5 (*always/almost always*), with greater numbers signifying an increased risk of problematic internet use. The sum of all individual item scores constituted the total score.

##### 3.1.3.3 Impulsivity

Trait impulsivity was measured using a validated French 22-item version (33) of the BIS-11 (34). Participants rated how often they thought or behaved in certain ways by selecting a response from a 4-point Likert scale ranging 1 (*rarely/never*) to 4 (*almost always/always*). Item scores were summed to obtain a total score for impulsivity.

#### 3.1.4 Analytic strategy

As in Study 1.

### 3.2 Results

Descriptive statistics (M and SD) as well as reliability estimates (α) for all constructs measured are reported in **Table 4**.

**Table 4:** Descriptive statistics and reliability for all variables from the French validation.

Variables	Switzerland		
	*M*	*SD*	*α*
IDL	49.35	21.54	.88
QDE	3.23	0.73	.96
QDE Wellbeing	3.14	0.84	.87
QDE Social Connectedness	2.94	0.88	.95
QDE Time and Efficiency	3.66	0.78	.94
Problematic internet use	21.48	7.02	.88
Emotional wellbeing	15.37	4.63	.87
Satisfaction with life	22.82	6.86	.89
Impulsivity	49.36	6.91	.70

IDL, Immersion in Digital Life; QDE, Quality of Digital Experience; QDE Wellbeing, Quality of Digital Experience (Wellbeing); QDE Social Connectedness, Quality of Digital Experience (Social Connectedness); QDE Time and Efficiency, Quality of Digital Experience (Time and Efficiency).

#### 3.2.1 IDLS

The CFA showed that the χ2 for the model was significant (χ2 = 42.17; df = 5; p <.001). This model did not show acceptable indicators of goodness of fit for CMIN/DF and RMSEA (CMIN/DF = 8.43; RMSEA = .11; GFI = .97; CFI = .98; SRMR = .03). To improve the model fit, one covariance was added (CMIN/DF = 3.47; RMSEA = .06; GFI = .99; CFI = .99; SRMR = .02). After adding the covariance, items 3 and 5 had loadings >.40 and <.70. As the loadings approached .70 (item 3 = .66 and item 5 = .69) no items were removed and one residual stayed in the final model (CMIN/DF = 3.47; RMSEA = .06; GFI = .99; CFI = .99; SRMR = .02). **Figure 2.1** in the **Supplementary Material** presents the final model. Internal consistency (α = .88) as well as split-half reliability of the IDLS (r =.74; ρ =.85; G = .83) were acceptable.

**Table 5** shows correlational analyses performed to assess convergent and discriminant validity. As expected, IDL correlated positively with QDE, QDE Wellbeing, Social connectedness and Time and efficiency. In addition, the anticipated positive correlation of IDL with problematic internet use was confirmed, suggesting adequate convergent validity of the IDLS. There was no association between IDL and satisfaction with life or emotional wellbeing. IDL correlated positively with impulsivity.

**Table 5:** Intercorrelations between IDL, QDE and subdimensions of QDE for the French validation.

Switzerland	IDL	QDE	QDE Wellbeing	QDE Social Connectedness	QDE Time and Efficiency
IDL	–				
QDE	.64**	–			
QDE Wellbeing	.59**	.86**	–		
QDE Social Connectedness	.64**	.92**	.76**	–	
QDE Time and Efficiency	.40**	.79**	.56**	.52**	–
Problematic internet use	.48**	.38**	.36**	.41**	.20**
Emotional wellbeing	-.00	.16**	.14**	.15**	.12**
Satisfaction with life	.06	.22**	.16**	.21**	.19**
Impulsivity	.08*	.05	.05	.10*	-.03

Correlations of IDL, QDE and subdimensions of QDE with relevant measures.

*p <.05; **p <.01.

IDL, Immersion in Digital Life; QDE, Quality of Digital Experience; QDE Wellbeing, Quality of Digital Experience (Wellbeing); QDE Social Connectedness, Quality of Digital Experience (Social Connectedness); QDE Time and Efficiency, Quality of Digital Experience (Time and Efficiency).

#### 3.2.2 QDES

The CFA showed that the χ2 for the model was significant (χ2 = 1107.17; df = 296; p <.001). This model did not show acceptable indicators of goodness of fit for GFI (CMIN/DF = 3.74; RMSEA = .07; GFI = .88; CFI = .94; SRMR = .05). To improve the model fit, we added covariances step by step starting with the highest and checking goodness of fit after every step. The first covariance was added (CMIN/DF = 3.63; RMSEA = .06; GFI = .88; CFI = .94; SRMR = .05). To improve GFI, a second covariance was added (CMIN/DF = 3.54; RMSEA = .06; GFI = .89; CFI = .94; SRMR = .05). To improve the model, a third covariance was added (CMIN/DF = 3.45; RMSEA = .06; GFI = .89; CFI = .94; SRMR = .05). After adding the third covariance, one item had loading >.40 and <.70. (item 18 = .67). GFI still did not meet the required standard, but the rest of the indicators met the requirements for goodness of fit, especially prioritized indicator: CFI and SRMR. No items were removed and three residuals stayed in the final model. **Figure 2.2** in the **Supplementary Material** presents the final model. Internal consistency as well as split-half reliability of the full QDES (α = .96; r = .94; ρ = .97; G = .97) as well as the Wellbeing (α = .87; r = .80; ρ = .89; G = .84), Social Connectedness (α = .95; r =.92; ρ = .96; G = .96) and Time and Efficiency (α = .94; r = .88; ρ = .94; G = .92) subscales were acceptable.

The correlational analysis in **Table 5** shows that QDE correlated with QDE Wellbeing, Social connectedness and Time and efficiency. The positive correlation between QDE and problematic internet use suggested convergent validity. QDE correlated positively with emotional wellbeing and satisfaction with life but there was no correlation with impulsivity.

## 4 Study 3: validation of the IDLS and QDES in Spanish

### 4.1 Materials and methods

#### 4.1.1 Participants

A sample of 1224 individuals representative in terms of age and gender of the population of Spain participated in the study. 429 participants were excluded for incorrectly answering one or more of three attention check questions included in the questionnaire. The analyses presented are based on a final sample of 795 participants after data exclusions. See **Table 1** for sample details.

#### 4.1.2 Procedure

Data collection was initiated on 17th October 2023 and completed on 20th October 2023. Mean study completion time was 15.29 minutes. The remaining procedure was identical to that of study 1.

##### 4.1.2.1 Translation of the IDLS and QDES

The IDLS and QDES were translated to Spanish following the same method and procedure as outlined for study 1. For step 5, feedback on the prefinal versions of the questionnaires was collected from two laypeople whose first language was Spanish. The final copies of the IDLS and QDES used in this study are provided in the **Supplementary Material**.

#### 4.1.3 Measures

Demographic details, IDL, QDE, emotional wellbeing and satisfaction with life were measured using Spanish versions of the demographic questionnaire, the IDLS, the QDES, the WHO-5 (35) and the SWLS (36), respectively. The Spanish SWLS was based on a 5-point Likert scale ranging from 1 (*strongly disagree*) to 5 (*strongly agree*) but was otherwise identical to the SWLS described in study 1. Please see the section “Measures” of study 1 for a description of the mentioned instruments used in this study.

##### 4.1.3.1 Demographic details

Gender was measured using a multiple-choice question as in study 1 and six response options (“male”, “female”, “non-binary”, “transgender”, “other (please describe)” and “prefer not to say”).

##### 4.1.3.2 Problematic internet use

The 15-item Generalized Problematic Internet Use Scale-2 (GPIUS-2) (37) was used to assess problematic internet use. This measure is composed of five subscales (Preference for online social interaction, Mood regulation, Negative outcomes, Cognitive preoccupation, and Compulsive internet use). Respondents indicated their level of agreement with 15 statements by rating them on a 6-point Likert-scale ranging from 1 (*strongly disagree*) to 6 (*strongly agree*). A total score was calculated by computing means of individual item scores, with higher scores indicating greater problematic internet use. Subscale scores are not reported in this study.

##### 4.1.3.3 Impulsivity

The 20-item UPPS−P Impulsive Behaviour Scale (UPPS-P) (38) was used to measure trait impulsivity. The measure is composed of five subscales (Lack of premeditation, Lack of perseverance, Sensation seeking, Negative urgency and Positive urgency). Participants indicated their agreement with 20 statements by selecting a response from a 4-point Likert scale ranging from 1 (*strongly agree*) to 4 (*strongly disagree*). The total score was computed by calculating the mean of individual item scores.

#### 4.1.4 Analytic strategy

As in Study 1.

### 4.2 Results

Descriptive statistics (M and SD) as well as reliability estimates (α) for all constructs measured are reported in **Table 6**.

**Table 6:** Descriptive statistics and reliability for all variables from the Spanish validation.

Variables	Spain		
	*M*	*SD*	*α*
IDL	45.15	20.00	.85
QDE	3.40	0.69	.96
QDE Wellbeing	3.24	0.83	.90
QDE Social Connectedness	3.21	0.81	.95
QDE Time and Efficiency	3.74	0.74	.94
Problematic internet use	3.60	0.95	.93
Emotional wellbeing	14.81	4.52	.88
Satisfaction with life	16.54	4.56	.89
Impulsivity	2.15	0.38	.83

IDL, Immersion in Digital Life; QDE, Quality of Digital Experience; QDE Wellbeing, Quality of Digital Experience (Wellbeing); QDE Social Connectedness, Quality of Digital Experience (Social Connectedness); QDE Time and Efficiency, Quality of Digital Experience (Time and Efficiency).

#### 4.2.1 IDLS

The CFA showed that the χ2 for the model was significant (χ2 = 60.99; df = 5; p <.001). This model did not show acceptable indicators of goodness of fit (CMIN/DF = 12.20; RMSEA = .12; GFI = .97; CFI = .97; SRMR = .03). To improve the model fit, one theoretically viable error covariance was added between items 3 and 5 (CMIN/DF = 2.68; RMSEA = .05; GFI = 1.00; CFI = 1.00; SRMR = .01). After adding this covariance, factor loading for item 3 dropped from .67 to .60, and factor loading for item 2 increased from .68 to .69; all other factor loadings were above .70 and the highest was item 4 (which increased from .80 to .83). **Figure 3.1** in the **Supplementary Material** presents the final model. Internal consistency (α = .85) as well as split-half reliability of the IDLS (r =.79; ρ =.89; G = .86) were acceptable.

**Table 7** shows the results of the correlational analyses performed to assess convergent and discriminant validity. As expected, IDL correlated positively with QDE, QDE Wellbeing, Social connectedness and Time and efficiency. The positive correlation between IDL and problematic internet use suggests convergent validity. IDL also correlated positively with impulsivity. However, there was no association between IDL and emotional wellbeing and satisfaction with life.

**Table 7:** Intercorrelations between IDL, QDE and subdimensions of QDE for the Spanish validation.

Spain	IDL	QDE	QDE Wellbeing	QDE Social Connectedness	QDE Time and Efficiency
IDL	–				
QDE	.59**	–			
QDE Wellbeing	.53**	.90**	–		
QDE Social Connectedness	.55**	.88**	.72**	–	
QDE Time and Efficiency	.46**	.83**	.61**	.58**	–
Problematic internet use	.46**	.47**	.45**	.48**	.30**
Emotional wellbeing	.00	.13**	.08*	.13**	.14**
Satisfaction with life	.03	.10**	.04	.12**	-10**
Impusivity	.13**	.09*	.10**	.12**	.01

Correlations of IDL, QDE and subdimensions of QDE with relevant measures.

*p <.05; **p <.01.

IDL, Immersion in Digital Life, QDE, Quality of Digital Experience, QDE Wellbeing, Quality of Digital Experience (Wellbeing), QDE Social Connectedness, Quality of Digital Experience (Social Connectedness), QDE Time and Efficiency, Quality of Digital Experience (Time and Efficiency).

#### 4.2.2 QDES

The CFA showed that the χ2 for the model was significant (χ2 = 1179.00; df = 296; p <.001). This model showed an acceptable fit, with the exception of GFI (CMIN/DF = 3.98; RMSEA = .06; GFI = .89; CFI = .95; SRMR = .05). To improve the model fit, two theoretically viable error covariances were added step by step in the first factor (Social connectedness) starting with the highest modification index and checking goodness of fit after every step. The first error covariance was added between items 6 and 11 (CMIN/DF = 3.85; RMSEA = .06; GFI = .90; CFI = .95; SRMR = .05). To improve GFI, a second covariance was added between items 12 and 15 (CMIN/DF = 3.72; RMSEA = .06; GFI = .90; CFI = .95; SRMR = .05). All factor loadings were between .71 and .86. **Figure 3.2** in the **Supplementary Material** presents the final model. Internal consistency as well as split-half reliability of the full QDES (α = .96; r =.92; ρ = .96; G = .96) as well as the Wellbeing (α = .90; r = .82; ρ = .91; G = .88), Social Connectedness (α = .95; r = .89; ρ = .94; G = .94) and Time and Efficiency (α = .94; r =.87; ρ = .93; G = .92) subscales were acceptable.

The correlational analysis in **Table 7** shows that QDE correlated positively with QDE Wellbeing, Social connectedness and Time and Efficiency. The anticipated positive correlation between QDE and problematic internet use suggests convergent validity. QDE correlated positively with emotional wellbeing and satisfaction with life but there was no relationship between QDE and impulsivity.

## 5 Study 4: validation of the IDLS and QDES in Polish

### 5.1 Materials and methods

#### 5.1.1 Participants

A sample of 1208 individuals representative in terms of age, gender, education and place of residence of the population of Poland participated in the study. Participants who incorrectly answered any of the attention check questions included in the study were excluded prior to data transfer by the survey company PBS who collected the data. As a result it is not possible to provide information on the number of excluded participants in Poland. See **Table 1** for sample details.

#### 5.1.2 Procedure

Data collection was initiated on 19th September 2023 and completed on 4th October 2023. Mean study completion time was 19.93 minutes. Recruitment of participants was undertaken by the recruitment platform PBS. The remaining procedure was identical to that of study 1.

##### 5.1.2.1 Translation of the IDLS and QDES

The IDLS and QDES were translated to Polish following the same method and procedure as outlined for study 1. For step 5, feedback on the prefinal versions of the questionnaires was collected from six laypeople whose first language was Polish. The final copies of the IDLS and QDES used in this study are provided in the **Supplementary Material**.

#### 5.1.3 Measures

Demographic details, IDL, QDE, emotional wellbeing, satisfaction with life, problematic internet use and impulsivity were measured using Polish versions of the demographic questionnaire, the IDLS, the QDES, the WHO-5 (39), the SWLS (40), the GPIUS-2 (41) and the BIS-11 (42) respectively. Please see the section “Measures” of study 1 for a description of the demographic questionnaire, the IDLS, the QDES, the WHO-5, SWLS and the BIS-11 and section “Measures” of study 3 for a description of the GPIUS-2.

##### 5.1.3.1 Demographic details

Gender was measured using a multiple-choice question as in study 1 and four response options (“male”, “female”, “non-binary” and “other”).

#### 5.1.4 Analytic strategy

The analytic strategy was identical to that used in study 1.

### 5.2 Results

Descriptive statistics (M and SD) as well as reliability estimates (α) for all constructs measured are reported in **Table 8**.

**Table 8:** Descriptive statistics and reliability for all variables from the Polish validation.

Variables	Poland		
	*M*	*SD*	*α*
IDL	51.74	19.98	.86
QDE	3.45	0.70	.96
QDE Wellbeing	3.47	0.80	.90
QDE Social Connectedness	3.15	0.86	.96
QDE Time and Efficiency	3.82	0.71	.93
Problematic internet use	3.13	1.13	.92
Emotional wellbeing	13.27	5.15	.91
Satisfaction with life	19.84	6.29	.90
Impulsivity	61.41	9.71	.81

IDL, Immersion in Digital Life; QDE, Quality of Digital Experience; QDE Wellbeing, Quality of Digital Experience (Wellbeing); QDE Social Connectedness, Quality of Digital Experience (Social Connectedness); QDE Time and Efficiency, Quality of Digital Experience (Time and Efficiency).

#### 5.2.1 IDLS

The CFA showed that the χ2 for the model was significant (χ2 = 30.99; df = 5; p <.001). This model did not show acceptable indicators of goodness of fit (CMIN/DF = 13.65; RMSEA = .10; GFI = .98; CFI = .98; SRMR = .03). One theoretically supported error covariance was then added between items 3 and 5, resulting in an excellent fit (CMIN/DF = 3.00; RMSEA = .04; GFI = 1.00; CFI = 1.00; SRMR = .01). Factor loadings in the final model were all ≥.70 or approached this figure in one case (item 3 = .69). **Figure 4.1** in the **Supplementary Material** presents the final model. Internal consistency (α = .86) as well as split-half reliability of the IDLS (r = .73; ρ =.84; G = .82) were acceptable.

**Table 9** shows the results of the correlational analyses performed to assess convergent and discriminant validity. As expected, IDL correlated positively with QDE, QDE Wellbeing, Social connectedness and Time and efficiency. The positive correlation between IDL and problematic internet use suggests convergent validity. IDL correlated positively with impulsivity; however, it was not associated with emotional wellbeing or satisfaction with life.

**Table 9:** Intercorrelations between IDL, QDE and subdimensions of QDE for the Polish validation.

Poland	IDL	QDE	QDE Wellbeing	QDE Social Connectedness	QDE Time and Efficiency
IDL	–				
QDE	.55*	–			
QDE Wellbeing	.50**	.88**	–		
QDE Social Connectedness	.52**	.93**	.77**	–	
QDE Time and Efficiency	.41**	.81**	.64**	.57**	–
Problematic internet use	.37**	.36**	.39**	.38**	.17**
Emotional wellbeing	-.00	.09**	.05	.09**	.10**
Satisfaction with life	-.01	.04	-.02	.04	.07**
Impulsivity	.10**	-.03	.04	.03	-.14**

Correlations of IDL, QDE and subdimensions of QDE with relevant measures.

*p <.05; **p <.01.

IDL, Immersion in Digital Life; QDE, Quality of Digital Experience; QDE Wellbeing, Quality of Digital Experience (Wellbeing), QDE Social Connectedness, Quality of Digital Experience (Social Connectedness), QDE Time and Efficiency, Quality of Digital Experience (Time and Efficiency).

#### 5.2.2 QDES

The CFA showed that the χ2 for the model was significant (χ2 = 1765.91; df = 290; p <.001). The initial model showed suboptimal fit (CMIN/DF = 7.33; RMSEA = .07; GFI = .87; CFI = .93; SRMR = .05). To improve the model, six theoretically justified error covariances were added step by step, starting with the highest modification indices. After each addition, the model fit improved incrementally. The final model showed an acceptable to good fit (CMIN/DF = 6.09; RMSEA = .07; GFI = .89; CFI = .95; SRMR = .05). All standardized factor loadings in the final model were ≥.70. Although the CMIN/DF exceeded the commonly recommended threshold of 5, this may be due to the large sample size, as this statistic is known to be overly sensitive in such cases and can lead to the false rejection of an otherwise acceptable model (23, 24). **Figure 4.2** in the **Supplementary Material** presents the final model. Internal consistency as well as split-half reliability of the full QDES (α = .96; r = .96; ρ = .98; G = .98) as well as the Wellbeing (α = .90; r = .82; ρ = .90; G = .87), Social Connectedness (α = .96; r = .94; ρ = .97; G = .97) and Time and Efficiency (α = .93; r = .89; ρ = .94; G = .93) subscales were acceptable.

The correlational analysis in **Table 9** shows that IDL correlated positively with QDE Wellbeing, Social connectedness and Time and efficiency. The positive correlation between QDE and problematic internet use suggests convergent validity of the QDES. QDE correlated positively with emotional wellbeing but was not associated with impulsivity or satisfaction with life.

## 6 Study 5: validation of the IDLS and QDES in Czech

### 6.1 Materials and methods

#### 6.1.1 Participants

A sample of 1216 participants representative in terms of age and gender of the population of Czechia participated in the study. 248 participants were excluded for incorrectly answering one or more of three attention check questions included in the questionnaire. The analyses presented are based on a final sample of 968 participants after data exclusions. See **Table 1** for sample details.

#### 6.1.2 Procedure

Data collection was initiated on 16th October 2023 and completed on 27th October 2023. Mean study completion time was 19.25 minutes. The remaining procedure was identical to that of study 1.

##### 6.1.2.1 Translation of the IDLS and QDES

The IDLS and QDES were translated to Czech following the same method and procedure as outlined for study 1. For step 5, feedback on the prefinal versions of the questionnaires was collected from five laypeople whose first language was Czech. The final copies of the IDLS and QDES used in this study are provided in the **Supplementary Material**.

#### 6.1.3 Measures

Demographic details, IDL, QDE, emotional wellbeing, satisfaction with life and impulsivity were measured using Czech versions of the demographic questionnaire, the IDLS, the QDES, the WHO-5 (43), the SWLS (44) and the UPPS-P (45). The UPPS-P used in this study was composed of four subscales (Lack of premeditation, Lack of perseverance, Sensation seeking and Urgency) rather than five as in the Spanish version (38). Please see the section “Measures” of study 1 for a description of the demographic questionnaire, the IDLS, the QDES, the WHO-5 and the SWLS. Please see section “Measures” of study 3 for a description of the UPPS-P.

##### 6.1.3.1 Demographic details

Gender was measured using a multiple-choice question as in study 1 and six response options (“male”, “female”, “non-binary”, “transgender”, “other (please describe)” and “prefer not to say”).

##### 6.1.3.2 Problematic internet use

The 20-item Internet Addiction Test (46) was used to assess problematic internet use. This measure required participants to indicate how frequently they displayed signs of problematic internet use on a 6-point Likert-scale ranging from 1 (*never*) to 6 (*always*). A total score was obtained by computing means of individual item scores, with higher scores indicating greater problematic internet use.

#### 6.1.4 Analytic strategy

As in Study 1.

### 6.2 Results

Descriptive statistics (M and SD) as well as reliability estimates (α) for all constructs measured are reported in **Table 10**.

**Table 10:** Descriptive statistics and reliability for all variables from the Czech validation.

Variables	Czechia		
	*M*	*SD*	*α*
IDL	52.08	21.86	.89
QDE	3.42	0.65	.94
QDE Wellbeing	3.39	0.78	.82
QDE Social Connectedness	3.26	0.78	.93
QDE Time and Efficiency	3.71	0.73	.89
Problematic internet use	3.36	1.37	.97
Emotional wellbeing	18.34	4.27	.66
Satisfaction with life	20.98	6.03	.89
Impulsivity	2.27	0.42	.84

IDL, Immersion in Digital Life; QDE, Quality of Digital Experience; QDE Wellbeing, Quality of Digital Experience (Wellbeing); QDE Social Connectedness; Quality of Digital Experience (Social Connectedness); QDE Time and Efficiency; Quality of Digital Experience (Time and Efficiency).

#### 6.2.1 IDLS

The CFA showed that the χ2 for the model was significant (χ2 = 28.86; df = 5; p <.001). Fit indices suggested an adequate fit to the data (CMIN/DF = 5.77; RMSEA = .07; GFI = .99; CFI = .99; SRMR = .02), and no modifications were necessary. All standardized factor loadings exceeded .70. **Figure 5.1** in the **Supplementary Material** presents the final model. Internal consistency (α = .89) as well as split-half reliability of the IDLS (r =.79; ρ =.88; G = .84) were acceptable.

**Table 11** shows the results of the correlational analyses performed to assess convergent and discriminant validity. IDL correlated positively with QDE, QDE Wellbeing, Social connectedness and Time and efficiency. The positive correlation between IDL and problematic internet use suggests convergent validity. IDL also correlated positively with impulsivity, satisfaction with life and emotional wellbeing.

**Table 11:** Intercorrelations between IDL, QDE and subdimensions of QDE for the Czech validation.

Czechia	IDL	QDE	QDE Wellbeing	QDE Social Connectedness	QDE Time and Efficiency
IDL	–				
QDE	.68**	–			
QDE Wellbeing	.59**	.82**	–		
QDE Social Connectedness	.67**	.92**	.69**	–	
QDE Time and Efficiency	.42**	.75**	.52**	.45**	–
Problematic internet use	.65**	.42**	.34**	.54**	.07**
Emotional wellbeing	.36**	.29**	.31**	.28**	.15**
Satisfaction with life	.25**	.23**	.13**	.23**	.18**
Impulsivity	.51**	.32**	.26**	.44**	.00

Correlations of IDL, QDE and subdimensions of QDE with relevant measures.

*p <.05; **p <.01.

IDL, Immersion in Digital Life; QDE, Quality of Digital Experience; QDE Wellbeing, Quality of Digital Experience (Wellbeing); QDE Social Connectedness, Quality of Digital Experience (Social Connectedness); QDE Time and Efficiency, Quality of Digital Experience (Timeand Efficiency).

#### 6.2.2 QDES

The CFA showed that the χ2 for the model was significant (χ2 = 1137.65; df = 201; p <.001). The initial model, including all original items, exhibited poor fit (CMIN/DF = 9.01; RMSEA = .09; GFI = .81; CFI = .84; SRMR = .08). As the addition of theoretically meaningful error covariances did not sufficiently improve model fit, four items with relatively low standardized factor loadings (.60–.66) were sequentially removed.

The decision to remove items 19, 1, 26, and 5 was further supported by the results of an Exploratory Factor Analysis (EFA) with Promax rotation (see **Table 1** in the **Supplementary Material** for an overview of the EFA results), which indicated that these items either loaded most strongly onto unintended factors or exhibited cross-loadings. Specifically, item 19 loaded on the Wellbeing factor (.63) instead of the presumed Time and efficiency factor (.23). Item 1 exhibited weak and similarly distributed loadings across all three factors (.25-.39), with the highest loading on the Wellbeing factor rather than the expected Social Connectedness factor. Item 26 showed similarly weak loadings on both the presumed Time and Efficiency factor (.41) and the Wellbeing factor (.29). Item 5 loaded on the Wellbeing factor (.64) as expected, nevertheless, this item showed only .60 loading in the final CFA model and its inclusion negatively affected model fit.

After item removal, the model showed improved but still suboptimal fit (CMIN/DF = 7.59; RMSEA = .08; GFI = .87; CFI = .89; SRMR = .05). Finally, five theoretically justified error covariances were added to the model, yielding acceptable fit indices (CMIN/DF = 5.66; RMSEA = .07; GFI = .90; CFI = .93; SRMR = .05). All standardized factor loadings in the final model were either above .70 or close to this threshold. **Figure 5.2** in the **Supplementary Material** presents the final model. Internal consistency as well as split-half reliability of the full QDES (α = .94; r =.89; ρ = .94; G = .94) as well as the Wellbeing (α = .82; r = .64; ρ = .78; G = .78), Social Connectedness (α = .93; r = .85, ρ = .92, G = .91) and Time and Efficiency (α = .89; r =.83; ρ = .91; G = .89) subscales were acceptable.

The correlational analysis in **Table 11** shows that IDL correlated positively with QDE Wellbeing, Social connectedness and Time and efficiency. The positive correlation between QDE and problematic internet use suggests convergent validity of the QDES. QDE correlated positively with emotional wellbeing and satisfaction with life. However, there was no relationship between QDE and impulsivity.

## 7 Discussion

The current study aimed to validate the Immersion in Digital Life Scale (IDLS) and the Quality of Digital Experience Scale (QDES) in five further languages: German, French, Spanish, Polish and Czech. To our knowledge, the IDLS and QDES are the only self-report measures of non-problematic DT use and experience that have been validated across six European countries. The instruments will for the first time provide psychological researchers with psychometrically robust tools to investigate indicators of digital wellbeing (i.e. the extent of DT use and experience of DT-related benefits in the areas of wellbeing, social connectedness and time and efficiency) across countries. This is significant as digital wellbeing research is particularly underdeveloped outside of the UK and US (69) and the applicability of DT-related studies conducted in anglophone nations to other European cultures is likely to be limited (70).

The availability of the IDLS and QDES in six different languages may enable researchers to uncover how prevalent positive digital experiences are in different cultural contexts (across and within countries) so that inequalities in digital wellbeing in Europe can be addressed more effectively and internationally. The application of the measures may also help shift the focus from the prevention or reduction of problematic digital engagement, still dominant across Europe, to the investigation of satisfying digital experiences and a more positive, normalising view of digital engagement. Furthermore, the instruments may provide an effective means for scholars and mental health professionals to assess the effectiveness of interventions aimed at improving digital wellbeing.

### 7.1 Scale summary

In all five of the studies presented, confirmatory factor analyses were performed for the IDLS and QDES, followed by an assessment of their reliability and convergent and discriminant validity. This analysis showed that the IDLS had a single factor structure, good psychometric properties, good reliability and associations with other measures in all five languages. The results therefore suggest that the 5-item scale is a valid and reliable measure of general digital immersion for use with French, German, Spanish, Polish and Czech speaking samples.

For the QDES, the German, French, Spanish and Polish language versions held the same factor structure as the original version validated for use in English (6). That is, three factors of Wellbeing (5 items), Social Connectedness (12 items) and Time and Efficiency (9 items). The German, French, Spanish and Polish language versions of the IDLS and QDES all had good psychometric properties, and the scales demonstrated excellent reliability and associations with other psychological constructs. For the Czech translation, the four items (19, 1, 26, and 5) with low psychometric fit were removed from the QDES to achieve good psychometric fit. Following this modification, the three-factor structure remained, consisting of Wellbeing (4 items), Social Connectedness (11 items) and Time and Efficiency (7 items). This version of the QDES demonstrated excellent reliability and associations with other psychological constructs. These results further confirm the ability of the IDLS and the QDES to effectively measure immersion and quality of digital experiences across multiple facets of life, independently of device or platform.

The poorer psychometric performance of four items of the Czech QDES may stem from translation challenges or cultural differences in how item content is perceived (71). Prior studies have shown that greater linguistic distance from English, the language of the original instrument, is associated with a higher number of non-equivalent items, particularly in Eastern European contexts (72). While a rigorous translation procedure was followed, the structural differences between English and Czech, as well as sociocultural context, may have contributed to subtle shifts in item meaning. Nevertheless, the overall structure of the QDES was preserved, supporting its robustness as a tool for assessing digital experiences across cultural and linguistic contexts. Moreover, the QDES and IDLS are not intended to be used as diagnostic tools and hence do not require any separate norms. However, removal of four items in the Czech version results in a difficulty to directly compare total scores from the Czech measure to the other country’s total scores without rescaling the raw scores into a common range (e.g., 0–1 or 1-100) by using simple linear transformation, or standardize scores using z-scores or t-scores. Researchers who intend to use the Czech QDES for cross cultural research should therefore ensure that these rescaling techniques are used prior to making comparisons.

### 7.2 Relationships to other measures

The positive associations between the IDLS and QDES observed across all countries replicate the relationships observed in the original questionnaire developed in the UK (6). Greater positive experience of DT is therefore associated with greater use of DT. In all countries, IDLS was positively associated with impulsivity, replicating Witowska et al.’s (6) findings with a UK sample. In a world in which DT is easily accessible, we speculate that this association may reflect a tendency for more impulsive individuals to preferentially use DT over non-digital tools because of their ever presence and the rapidity with which they can provide access to entertainment and communication.

The relationship between IDLS and QDES and measures of emotional wellbeing and satisfaction with life showed some variation across the five studies. Except in Germany, greater digital social connectedness (QDES Social connectedness) was associated with greater emotional wellbeing. Overall, quality of digital experience was also positively related to satisfaction with life in all samples except the Polish sample. In most countries, greater experiences of time saving and efficiency as a result of DT were also associated with better emotional wellbeing, suggesting that labor and time saving resulting from DT has positive benefits. Together, these findings confirm previous observations that DT use may enhance social relationships (47, 48) and improve wellbeing (49, 50) and suggest that having a positive experience of using DT may contribute to an overall sense of satisfaction with life.

Although our findings show that positive experiences of DT are broadly related to positive wellbeing, there were some cross-cultural differences in the relationships observed. Differential relationships between QDES and measures of emotional wellbeing and satisfaction with life may reflect cross-cultural differences in the design and use of digital medias in different territories (51), differing levels of digital literacy (52), differing social norms related to leisure and work (52, 53) and critically differing experiences of gratification from DT use (54).

These cross-cultural differences highlight the importance of developing and validating specific tools for specific populations when assessing the experience and impact of DT on health and wellbeing. Furthermore, they demonstrate that caution should be taken when applying evidence of the impact of DT use on health and wellbeing from one culture to other cultures. Future research should therefore prioritize understanding how and why culture affects our experiences of DT use and its subsequent impacts on health and wellbeing.

## 8 Limitations and future research

Whilst the current findings demonstrate relationships between DT immersion and experience and outcomes such as satisfaction with life and emotional wellbeing, the cross-sectional nature of these studies does not enable causal direction to be assessed. It therefore remains unclear whether, for example, the positive associations between quality of digital experience and satisfaction with life reflect positive DT experiences improving overall life satisfaction, or whether having an overall positive life experience predisposes individuals to positive digital experiences. Future research should therefore seek to understand how DT may act as a mediator or moderator to health and wellbeing across cultures. Moreover, the test-retest procedure has not been conducted, hence we cannot assume the stability of the measured constructs over time. Although the procedure followed to translate the IDLS and QDES was rigorous, the translated measures were evaluated by varying numbers of laypeople (native speakers of the target languages) in studies 1 to 5, which may affect the face validity of our questionnaires. In addition, due to the use of self-report instead of objective measures in our study, we recommend conducting experimental studies with behavioural measures to re-test the validity of the QDES and IDLS.

As in Witowska et al. (6), the current studies included samples which were representative of their country’s population in terms of age and gender, suggesting that the tools developed are valid for general population use. However, at present, validations of the IDLS and QDES are limited to European samples. To enable the scales to be employed in the full global context it is essential that they are culturally adapted and validated for use in the broad range of languages used in Southeast Asia, Africa, the Middle East and South America. Recent evidence from Southeast Asian contexts suggests that digital media are domesticated differently across societies and that it is important to avoid stereotyping users as digital natives (55). Furthermore, research conducted on teachers suggests that wellbeing practices such as mindfulness and gratitude require culturally sensitive adaptation even within broadly East Asian settings (56). Collectively this highlights the urgent needs to how the constructs of digital immersion and the quality of digital experience interact with local professional cultures, wellbeing initiatives, and institutional norms in countries such as Thailand, the Philippines, Vietnam, Indonesia, Malaysia, and Singapore and further afield. Doing so will enable the consideration of regional factors such as high mobile-only internet usage, inconsistent connectivity, collectivist cultural norms, and differences in the widespread use of platforms like Facebook, LINE, WhatsApp, and institution-specific learning systems, which could influence how items are interpreted and how scores are distributed. Such work will enable the development of culturally tailored and relevant policies to aid digital wellbeing across the world.

Furthermore, the tools have not been validated for use with specific populations who may experience particular barriers to DT use, or harms and benefits from its use (e.g. individuals with physical or intellectual difficulties). In addition, the questionnaires have only been validated in adult populations and we did not consider age, gender or other socioeconomic variables as potential moderators. We believe that the above-mentioned factors can act as moderators that may lead to differential outcomes across populations, potentially altering the strength or direction of observed effects. Future research should therefore seek to measure immersion in and experience of DTs in marginalized populations.

## 9 Conclusion

The current studies validated the IDLS and QDES in five further languages: German, French, Spanish, Polish and Czech. These validated tools enable measurement of our immersion in digital life and the quality of our digital experiences independent of devices or platforms, enabling assessment of the broad ways in which we use and experience DT. Furthermore, by being device and platform independent, these measures are able to withstand the rapidity with which DT devices, platforms and uses develop over time. The measures allow us also to assess DT use as a whole as well as the often-overlooked positive consequences of DT in everyday life. Understanding and measuring the positive impact of DT is crucial for the promotion of digital wellbeing and to develop and evaluate effective interventions aimed at increasing fulfilling digital experiences or reducing negative ones.

Furthermore, the instruments may provide an effective means for scholars and mental health professionals to assess the effectiveness of interventions aimed at improving digital wellbeing. Specifically, the IDLS and QDES can be used by individuals to assist them in self-evaluating their own quality of digital experiences by identifying the areas of life where digital technology is bringing them benefit and the areas where it is not. This information may assist individuals in adjusting their digital practices to achieve better outcomes. The IDLS and QDES will also enable policy makers and regulators to monitor the experiences of evolving digital technologies at a population level, therefore enabling the identification of trends over time. This type of large-scale longitudinal surveillance is critical to developing evidence-based policies aimed to regulate digital technology in the future. Finally, although the IDLS and QDES were not developed for clinical purposes, future research should explore their utility as a screening measure to identify individuals at risk of developing conditions associated with excessive digital technology use.

## Data Availability

The datasets supporting the Confirmatory Factor Analyses conducted in Studies 1–5 can be found in the online repository: https://osf.io/s4nf6/overview. The raw data covering the remaining variables analyzed in the study will be made available by the corresponding author upon request, without undue reservation.

## References

[1] Kushlev K Leitao MR . The effects of smartphones on well-being: theoretical integration and research agenda. Curr Opin Psychol. (2020) 36:77–82. doi: 10.1016/j.copsyc.2020.05.001, PMID: 32563707

[2] Nguyen MH . Managing social media use in an “always-on” society: exploring digital wellbeing strategies that people use to disconnect. Mass Commun Soc. (2021) 24:795–817. doi: 10.1080/15205436.2021.1979045

[3] Shaw H Ellis DA Geyer K Davidson BI Ziegler FV Smith A . Quantifying smartphone “use”: Choice of measurement impacts relationships between “usage” and health. TMB. (2020) 1(2). doi: 10.1037/tmb0000022

[4] Müller H Gove JL Webb JS Cheang A . (2015). Understanding and comparing smartphone and tablet use: Insights from a large-scale diary study, in: OzCHI '15: The Annual Meeting of the Australian Special Interest Group for Computer Human Interaction Parkville VIC Australia December 7 - 10, 2015, Parkville, VIC, Australia: Association for Computing Machinery, New York NY United States: Association for Computing Machinery. pp. 427–36. doi: 10.1145/2838739.2838748

[5] Ytre-Arne B . Media Use in Digital Everyday Life. Leeds: Emerald Publishing Limited (2023) p. 1–16.

[6] Witowska J Schoetensack C Ogden R Goncikowska K Wittmann M Černohorská V . New measurements of digital technology use: The Immersion in Digital Life and Quality of Digital Experience Scales. Front Psychiatry. (2025) 16:1595536. doi: 10.3389/fpsyt.2025.1595536, PMID: 40661888 PMC12257311

[7] Meerkerk G-J Van Den Eijnden RJJM Vermulst AA Garretsen HFL . The Compulsive Internet Use Scale (CIUS): Some psychometric properties. Cyberpsychol. Behav. (2009) 12:1–6. doi: 10.1089/cpb.2008.0181, PMID: 19072079

[8] Jiménez Rodríguez V Alvarado Izquierdo JM Llopis Pablos C . Validación de un cuestionario diseñado para medir frecuencia y amplitud de uso de las TIC. Edutec. Rev Electrónica Tecnología Educativa. (2017) 61:a368. doi: 10.21556/edutec.2017.61.949

[9] Harris B Regan T Schueler J Fields SA . Problematic mobile phone and smartphone use scales: A systematic review. Front Psychol. (2020) 11:672. doi: 10.3389/fpsyg.2020.00672, PMID: 32431636 PMC7214716

[10] Elphinston RA Gullo MJ Connor JP . Validation of the Facebook addiction questionnaire. Pers. Individ. Differ. (2022) 195:111619. doi: 10.1016/j.paid.2022.111619

[11] Fischer T Reuter M Riedl R . The Digital Stressors Scale: Development and validation of a new survey instrument to measure digital stress perceptions in the workplace context. Front Psychol. (2021) 12:607598. doi: 10.3389/fpsyg.2021.607598, PMID: 33776836 PMC7994533

[12] Yıldırım Şimşek K Çevik H . The development and validation of the Leisure Internet Usage Scale (LIUS). Leis. Stud. (2024) 43:76–88. doi: 10.1080/02614367.2023.2191978

[13] Bock BC Lantini R Thind H Walaska K Rosen RK Fava JL . The mobile phone affinity scale: enhancement and refinement. JMU. (2016) 4:e134. doi: 10.2196/mhealth.6705, PMID: 27979792 PMC5200845

[14] Griffioen N Van Rooij M Lichtwarck-Aschoff A Granic I . Toward improved methods in social media research. TMB. (2020) 1. doi: 10.1037/tmb0000005 PMC7013654 32012075

[15] Beaton DE Bombardier C Guillemin F Ferraz MB . Guidelines for the process of cross-cultural adaptation of self-report measures. Spine. (2000) 25:3186–91. doi: 10.1097/00007632-200012150-00014, PMID: 11124735

[16] Gürtler D Rumpf HJ Bischof A Kastirke N Meerkerk GJ John U . Psychometrische Eigenschaften und Normierung der deutschen Version der Compulsive Internet Use Scale (CIUS). Diagnostica. (2015) 61:210–21. doi: 10.1026/0012-1924/a000127

[17] WHO Collaborating Centre in Mental Health . WHO (Fünf) Fragebogen zum Wohlbefinden (1998). Available online at: https://www.psykiatri-regionh.dk/who-5/Documents/WHO5_German.pdf (Accessed May 6, 2025).

[18] Glaesmer H Grande G Braehler E Roth M . The German version of the Satisfaction with Life Scale (SWLS): Psychometric properties, validity, and population-based norms. Eur J Psychol Assess. (2011) 27:127–32. doi: 10.1027/1015-5759/a000058

[19] Preuss UW Rujescu D Giegling I Watzke S Koller G Zetzsche T . Psychometrische Evaluation der deutschsprachigen Version der Barratt-Impulsiveness-Skala. Nervenarzt. (2008) 79:305–19. doi: 10.1007/s00115-007-2360-7, PMID: 18210044

[20] Kline R . “Convergence of structural equation modeling and multilevel modeling”. In: Williams M Vogt WP , editors. The SAGE Handbook of Innovation in Social Research Methods. SAGE Publications Ltd, London (2011). p. 562–89.

[21] Kenny DA Kaniskan B McCoach DB . The performance of RMSEA in models with small degrees of freedom. Sociol Methods Res. (2015) 44:486–507. doi: 10.1177/0049124114543236

[22] Chen FF . Sensitivity of goodness of fit indexes to lack of measurement invariance. Struct Equ. Model. (2007) 14:464–504. doi: 10.1080/10705510701301834

[23] Brown TA . Confirmatory factor analysis for applied research. New York: The Guilford Press (2015). p. 76.

[24] Kyriazos TA . Applied psychometrics: sample size and sample power considerations in factor analysis (EFA, CFA) and SEM in general. Psychol. (2018) 9:2207–30. doi: 10.4236/psych.2018.98126

[25] Sharma S Mukherjee S Kumar A Dillon WR . A simulation study to investigate the use of cutoff values for assessing model fit in covariance structure models. J Bus. Res. (2005) 58:935–43. doi: 10.1016/j.jbusres.2003.10.007

[26] Hu LT Bentler PM . Cutoff criteria for fit indexes in covariance structure analysis: Conventional criteria versus new alternatives. Struct Equ. Modeling. (1999) 6:1–55. doi: 10.1080/10705519909540118

[27] Kyndt E Onghena P . “The integration of work and learning: Tackling the complexity with structural equation modelling”. In: Harteis C Rausch A Seifried J , editors. Discourses on Professional Learning: on the Boundary Between Learning and Working. Springer Netherlands, Dordrecht (2014). p. 255–91.

[28] McDonald RP . Path analysis with composite variables. Multivar. Behav Res. (1996) 31:239–70. doi: 10.1207/s15327906mbr3102_5, PMID: 26801458

[29] Nunnally JC . Psychometric Theory (2nd ed.). New York: McGraw-Hill (1978).

[30] WHO Collaborating Centre in Mental Health . Indice (en cinq points) de bien-être de l’OMS (1999). Available online at: https://www.psykiatri-regionh.dk/who-5/Documents/WHO5_French.pdf (Accessed May 12, 2025).

[31] Blais MR Vallerand RJ Pelletier LG Brière NM . L'échelle de satisfaction de vie: Validation canadienne-française du "Satisfaction with Life Scale." [The satisfaction scale: Canadian-French validation of the Satisfaction with Life Scale. Can J Behav Sci / Rev Can Des Sci du comportement. (1989) 21:210–23. doi: 10.1037/h0079854

[32] Laconi S Urbán R Kaliszewska-Czeremska K Kuss DJ Gnisci A Sergi I . Psychometric evaluation of the Nine-Item Problematic Internet Use Questionnaire (PIUQ-9) in nine European samples of internet users. Front Psychiatry. (2019) 10:136. doi: 10.3389/fpsyt.2019.00136, PMID: 30984037 PMC6448041

[33] Gelinas S Balbinotti MAA Labonte SH . Factor analysis of French translation of the Barratt impulsiveness scale (bis-11). Saúde Desenvolv. Hum. (2015) 3:55–67. doi: 10.18316/2317-8582.15.5

[34] Patton JH Stanford MS Barratt ES . Data from: barratt impulsiveness scale-11 (BIS-11) [Database record]. APA PsycTests. (1995). doi: 10.1037/t05661-000

[35] WHO Collaborating Centre in Mental Health . OMS (cinco) Indice de Bienestar (versión 1998) (1998). Available online at: https://www.psykiatri-regionh.dk/who-5/Documents/WHO5_Spanish.pdf (Accessed May 12, 2025).

[36] Atienza FL Pons D Balaguer I García-Merita M . Propiedades psicométricas de la Escala de Satisfacción con la Vida en adolescentes. Psicothema. (2000) 12:314–9.

[37] Gámez-Guadix M Orue I Calvete E . Evaluation of the cognitive-behavioral model of generalized and problematic Internet use in Spanish adolescents. Psicothema. (2013) 25:299–306. doi: 10.7334/psicothema2012.274, PMID: 23910742

[38] Cándido A Orduña E Perales JC Verdejo-García A Billieux J . Validation of a short Spanish version of the UPPS-P impulsive behaviour scale. Trastor. Adict. (2012) 14:73–8. doi: 10.1016/S1575-0973(12)70048-X

[39] WHO Collaborating Centre in Mental Health . Wskaźniki Dobrego Samopoczucia (WHO-5) . Available online at: https://www.psykiatri-regionh.dk/who-5/Documents/WHO5_Polish.pdf (Accessed May 12, 2025).

[40] Jankowski KS . Is the shift in chronotype associated with an alteration in well-being? Biol Rhythm Res. (2015) 46:237–48. doi: 10.1080/09291016.2014.985000

[41] Balcerowska JM Bereznowski P . The Generalized Problematic Internet Use Scale 2 in a Polish sample: psychometric validation and relationship with specific Internet-related disorders and psychosocial functioning. Curr Issues Personal. Psychol. (2023) 11:228–39. doi: 10.5114/cipp/151869, PMID: 38014388 PMC10654343

[42] Grzesiak M Beszłej JA Szechiński M . Skala impulsywności barratta. Postępy Psychiatrii i Neurologii. (2008) 17:61–4.

[43] WHO Collaborating Centre in Mental Health . WHO-5 Index emoční pohody (verze 1998) (1998). Available online at: https://www.psykiatri-regionh.dk/who-5/Documents/WHO5_Czech.pdf (Accessed May 12, 2025).

[44] Lewis CA Shevlin ME Smékal V Dorahy MJ . Factor structure and reliability of a Czech translation of the satisfaction with life scale among Czech University students. In Stud Psychol. (1999) 41(3):239–43.

[45] Linhartová P Širůček J Bartecek R Theiner P Jeřábková B Rudišinová D . České verze sebeposuzovacích modelů impulzivity Barrattovy škály a škály UPPS-P a jejich psychometrické charakteristiky. [Czech versions of impulslvity self-report scales the Barratt Scale and the UPPS-P Scale and their psychometric properties. Ceska Slov. Psychiatr. (2017) 113:149–57.

[46] Suchá J Dolejš M Pipová H Maierová E Cakirpaloglu P . Hraní digitálních her českými adolescenty. Olomouc: Univerzita Palackého v Olomouci (2018).

[47] Dredge R Schreurs L . Social media use and offline interpersonal outcomes during youth: A systematic literature review. Mass Commun Soc. (2020) 23:885–911. doi: 10.1080/15205436.2020.1810277

[48] Tammisalo K Rotkirch A . Effects of information and communication technology on the quality of family relationships: A systematic review. J Soc Pers. Relatsh. (2022) 39:2724–65. doi: 10.1177/02654075221087942

[49] Marciano L Lin J Sato T Saboor S Viswanath K . Does social media use make us happy? A meta-analysis on social media and positive well-being outcomes. SSM - Ment Health. (2024) 6:100331. doi: 10.1016/j.ssmmh.2024.100331

[50] Cuihong L Chengzhi Y . The impact of internet use on residents’ subjective well-being: An empirical analysis based on national data. SSIC. (2019) 40:106–28. doi: 10.1080/02529203.2019.1674039

[51] Proctor RW Nof SY Yih Y Balasubramanian P Busemeyer JR Carayon P . Understanding and improving cross-cultural decision making in design and use of digital media: A research agenda. Int J Hum.-Comput. Interact. (2011) 27:151–90. doi: 10.1080/10447318.2011.537175

[52] Hamutoglu NB Gemikonakli O De Raffaele C Gezgin DM . Comparative cross-cultural study in digital literacy. Eurasian J Educ Res. (2020) 88:121–48. doi: 10.14689/ejer.2020.88.6

[53] Jolly C Shivani F . Cross-cultural perspectives on digital media and well-being: A narrative review. IJSSR. (2024) 12:108–10. doi: 10.5005/jp-journals-11001-0069

[54] Deng T Vargas-Bianchi L Mensa M . Cross-cultural comparison of TikTok uses and gratifications. Behav Inf Technol. (2024) 43:3047–59. doi: 10.1080/0144929X.2023.2270534

[55] Lo NP . Revolutionising language teaching and learning via digital media innovations. In: In Learning Environment and Design: Current and Future Impacts. Springer Singapore, Singapore (2020). p. 245–61.

[56] Lo NP Punzalan CH . The impact of positive psychology on language teachers in higher education. J Univ Teach Learn Practice. (2025) 22:1–9. doi: 10.53761/5ckx2h71

[57] Ozimek P Brailovskaia J Bierhoff HW . (2023). Active and passive behavior in social media: Validating the Social Media Activity Questionnaire (SMAQ). Telemat. Inform. Rep.. 10:100048. doi: 10.1016/j.teler.2023.100048

[58] Zarraluqui López S Martínez M Pastor MA . (2021). Development and validation of a questionnaire in Spanish for evaluating Facebook use. Curr Psychol. 40:2453–2461. doi: 10.1007/s12144-019-00171-7

[59] Uram P Skalski-Bednarz SB . (2024). Social media escapism in Poland: Adaptation of a measure and its relationship with thought suppression and mental health. Psychol Rep. 332941241269552. doi: 10.1177/00332941241269552, PMID: 39151126

[60] König R Seifert A . (2023). Internet usage, frequency and intensity in old age during the COVID-19 pandemic—a case study for Switzerland. Front Sociol. 8:1268613. doi: 10.3389/fsoc.2023.1268613, PMID: 37954497 PMC10639129

[61] Baggio S Iglesias K Berchtold A Suris JC . (2016). Measuring internet use: comparisons of different assessments and with internet addiction. Addict. Res. Theory. 25:114–120. doi: 10.1080/16066359.2016.1206083

[62] Brosch A . (2017). The need for popularity and Facebook usage among Czech and Polish young adults. TNER. 50:109–122. doi: 10.15804/tner.2017.50.4.09

[63] Juhaňák L Zounek J Záleská K Bárta O Vlčková K . (2019). The relationship between students’ ICT use and their school performance: Evidence from PISA 2015 in the Czech Republic. Orb. Sch.. 12:37–64. doi: 10.14712/23363177.2018.292

[64] Boer M van den Eijnden RJJM Finkenauer C Boniel-Nissim M Marino C Inchley J . (2022). Cross-national validation of the social media disorder scale: findings from adolescents from 44 countries. Addiction. 117:784–795. doi: 10.1111/add.15709, PMID: 34605094 PMC7614030

[65] Škařupová K Ólafsson K Blinka L . (2015). Excessive internet use and its association with negative experiences: Quasi-validation of a short scale in 25 European countries. Comput. Human Behav.. 53:118–123. doi: 10.1016/j.chb.2015.06.047

[66] Lopez-Fernandez O Kuss DJ Pontes HM Griffiths MD Dawes C Justice LV . (2018). Measurement invariance of the short version of the Problematic Mobile Phone Use Questionnaire (PMPUQ-SV) across eight languages. Int. J. Environ. Res. Public Health. 15:1213. doi: 10.3390/ijerph15061213, PMID: 29890709 PMC6025621

[67] Koronczai B Urbán R Kökönyei G Paksi B Papp K Kun B . (2011). Confirmation of the three-factor model of problematic internet use on off-line adolescent and adult samples. Cyberpsychology, Behavior, and Social Networking. 14(11):657–664. doi: 10.1089/cyber.2010.0345, PMID: 21711129 PMC3225044

[68] Ancis JR . (2020). The age of cyberpsychology: An overview. Technology, Mind, and Behavior. 1. doi: 10.1037/tmb0000009

[69] Al-Mansoori RS Al-Thani D Ali R . (2023). Designing for digital wellbeing: From theory to practice a scoping review. Human Behavior and Emerging Technologies. 1:9924029. 10.1155/2023/9924029

[70] Papadopoulos N Cleveland M . (2023). An international and cross-cultural perspective on ‘the wired consumer’: The digital divide and device difference dilemmas. Journal of Business Research. 156:113473. 10.1016/j.jbusres.2022.113473

[71] Bagheri Z Chamanpara P Jafari P Balhara YPS Arya S Ransing R . (2022). Cross-cultural measurement invariance of the Quality of Life Enjoyment and Satisfaction Questionnaire-Short form across ten countries: the application of Bayesian approximate measurement invariance. BMC Psychology. 10:160. 10.1186/s40359-022-00864-y, PMID: 35751087 PMC9229907

[72] Scott NW Fayers PM Bottomley A Aaronson NK De Graeff A Groenvold M . (2006). Comparing translations of the EORTC QLQ-C30 using differential item functioning analyses. Quality of Life Research. 15:1103–1115. 10.1007/s11136-006-0040-x, PMID: 16900290

[73] Shaw H Ellis DA Ziegler FV . (2018). The technology integration model (TIM): Predicting the continued use of technology. Computers in Human Behavior. 83:204–214.

[74] Potter J McDougall J . (2017). Digital Media, Culture and Education: Theorising Third Space Literacies. Palgrave MacMillan, Basingstoke.

[75] Harari GM Vaid SS Müller SR Stachl C Marrero Z Schoedel R . (2020). Personality sensing for theory development and assessment in the digital age. European Journal of Personality. 34(5):649–669. doi: 10.1002/per.2273

