# Supplementary material for: Validation of the immersion in digital life and quality of digital experience scales in German, French, Spanish, Polish and Czech
Source: Front Psychiatry. 2026 Feb 19;16:1645260. doi: 10.3389/fpsyt.2025.1645260 (PMC12960581; doi:10.3389/fpsyt.2025.1645260)
Supplement: Supplementary file 1 [file Supplementaryfile1.docx]

Supplementary Material

# Supplementary Figures


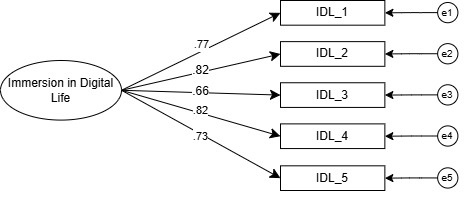


**Figure 1.1.** Final standardized model of the German IDLS


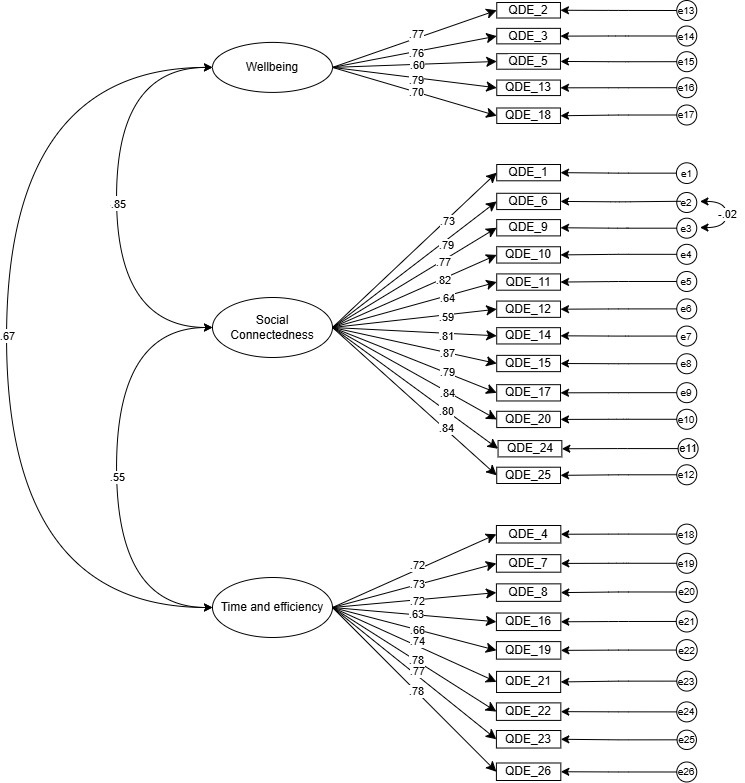


**Figure 1.2.** Final standardized model of the German QDES


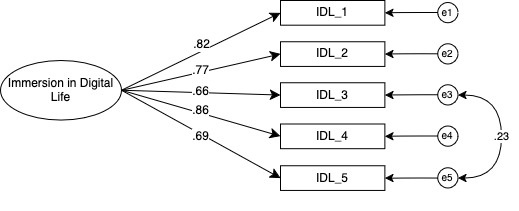


**Figure 2.1.** Final standardized model of the French IDLS


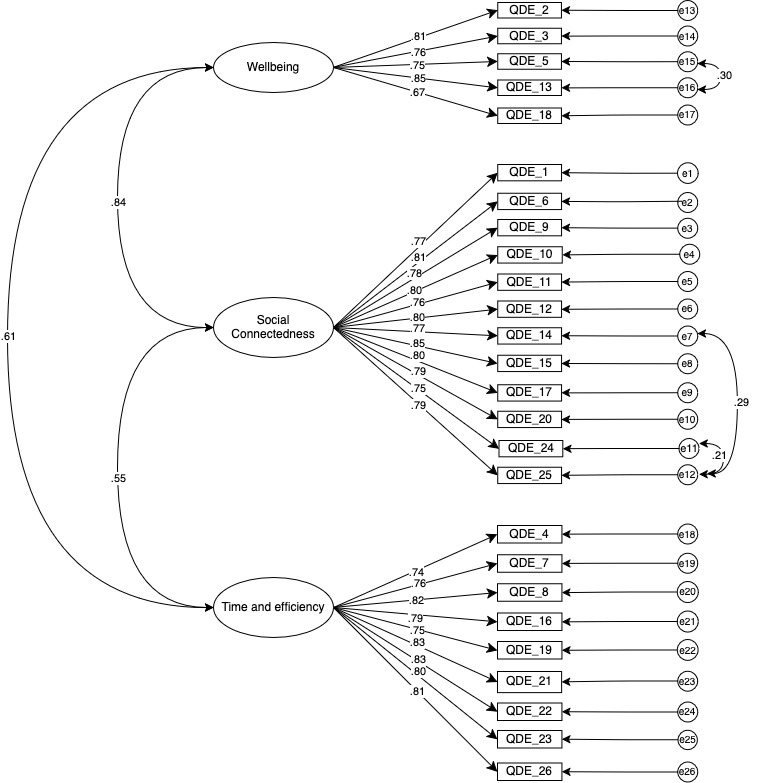


**Figure 2.2.** Final standardized model of the French QDES


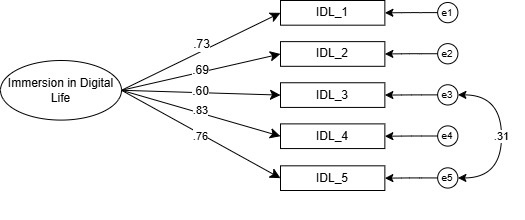


**Figure 3.1.** Final standardized model of the Spanish IDLS


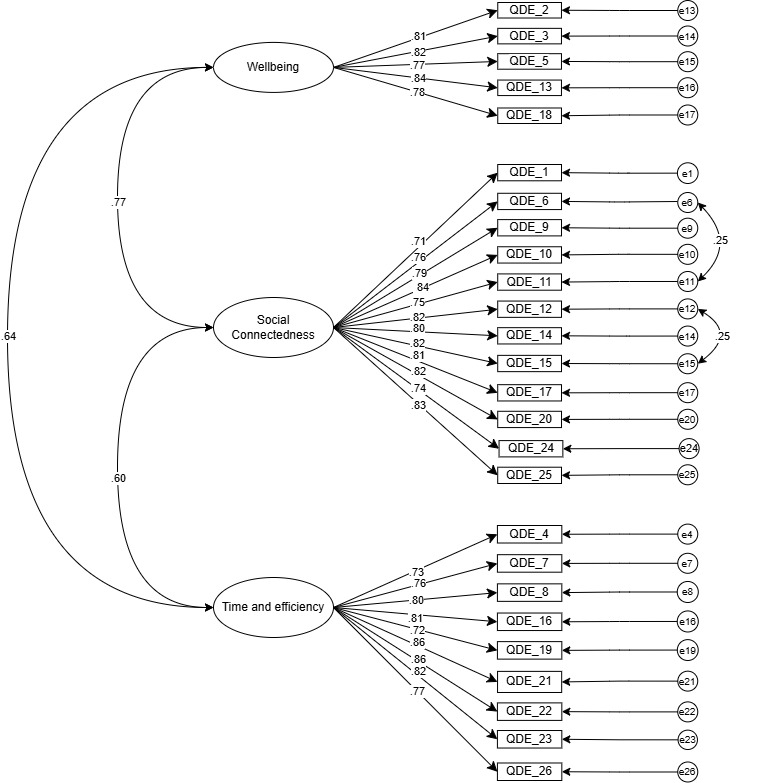


**Figure 3.2.** Final standardized model of the Spanish QDES


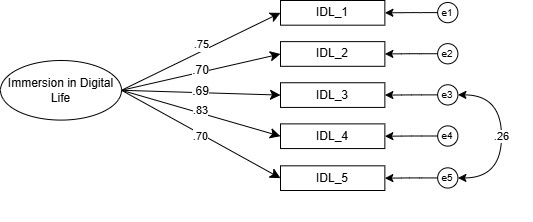


**Figure 4.1.** Final standardized model of the Polish IDLS


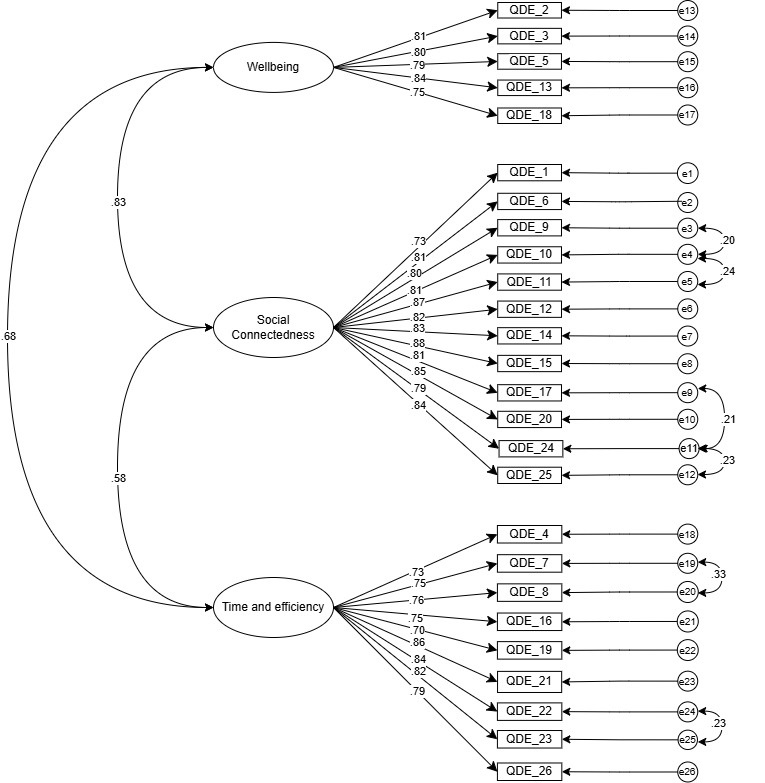


**Figure 4.2.** Final standardized model of the Polish QDES


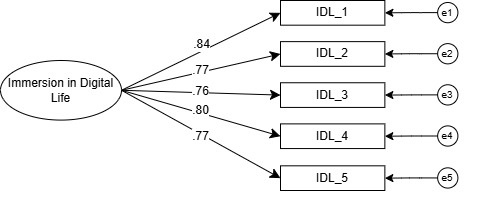


**Figure 5.1.** Final standardized model of the Czech IDLS


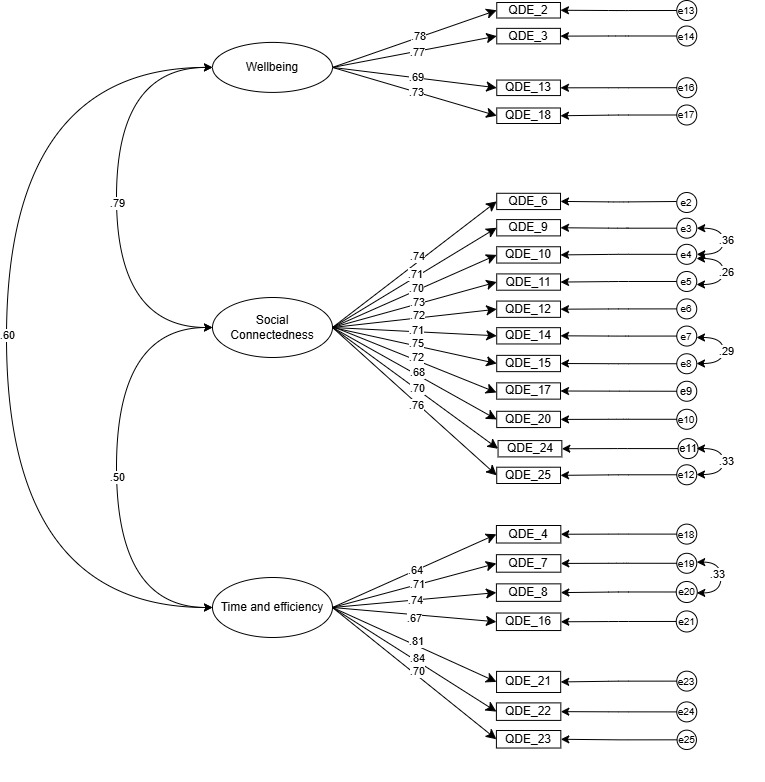


**Figure 5.2.** Final standardized model of the Czech QDES

# Supplementary Table

|  | QDE Wellbeing | QDE Social Connectedness | QDE Time and Efficiency |
| --- | --- | --- | --- |
| Item 24 | -.211 | .963 | -.095 |
| Item 25 | -.002 | .845 | -.079 |
| Item 10 | -.033 | .793 | -.013 |
| Item 9 | -.150 | .783 | .162 |
| Item 15 | -.039 | .753 | .109 |
| Item 11 | .059 | .718 | .002 |
| Item 14 | .036 | .717 | .027 |
| Item 6 | .145 | .657 | -.001 |
| Item 12 | .174 | .654 | -.077 |
| Item 17 | .159 | .604 | .053 |
| Item 20 | .227 | .510 | .048 |
| Item 8 | -.129 | .068 | .866 |
| Item 22 | -.018 | -.010 | .848 |
| Item 21 | .130 | -.159 | .810 |
| Item 23 | -.206 | .159 | .798 |
| Item 7 | .015 | .060 | .750 |
| Item 16 | .011 | -.058 | .749 |
| Item 4 | .216 | -.103 | .609 |
| Item 26 | .287 | .117 | .414 |
| Item 18 | .864 | -.005 | -.089 |
| Item 3 | .829 | .006 | -.030 |
| Item 2 | .733 | .149 | -.082 |
| Item 5 | .641 | -.120 | .205 |
| Item 19 | .628 | -.053 | .226 |
| Item 13 | .486 | .409 | -.143 |
| Item 1 | .388 | .245 | .269 |

*Note.* Total variance explained = 59.06%.

**Supplementary Table 1.** Results of the Exploratory Factor Analysis (EFA) for the Czech version of the QDES presented in study 5.

# Supplementary Data

**German version of the IDLS**

Fragebogen zum Ausmaß des digitalen Alltagslebens

Die folgenden Fragen beziehen sich auf das Ausmaß, in dem Sie digitale Technologien in Ihrem Leben nutzen. Für die Zwecke dieses Fragebogens definieren wir digitale Technologie als **Geräte, Systeme, Dienste, Daten oder Prozesse, die in irgendeiner Weise digitale Informationen nutzen, z. B. Computer, Tablets, Mobiltelefone oder Smart-TVs und andere Geräte**. Diese Technologien können für verschiedene Zwecke wie Kommunikation, Unterhaltung, Arbeit, Bildung und alltägliche Aufgaben verwendet werden.

Bitte lesen Sie jede einzelne Frage und antworten Sie so ehrlich wie möglich. Denken Sie dabei an Ihre persönlichen Erfahrungen. Bitte ziehen Sie den Schieberegler unter jeder Frage, um **das Ausmaß** der Digitalität der jeweiligen Lebensaktivität anzugeben. Es gibt keine richtigen oder falschen Antworten, wir möchten Ihre persönlichen Erfahrungen wissen.

1. In welchem Ausmaß werden Ihre sozialen Beziehungen digital geführt?

Überhaupt nicht digital Vollständig digital

___________________________________________________________________

1. In welchem Ausmaß ist Ihre Kommunikation mit der Familie digital?

Überhaupt nicht digital Vollständig digital

___________________________________________________________________

1. In welchem Ausmaß sind die Aktivitäten, denen Sie in Ihrer Freizeit nachgehen, digital?

Überhaupt nicht digital Vollständig digital

___________________________________________________________________

1. In welchem Ausmaß ist Ihre Kommunikation mit Freunden digital?

Überhaupt nicht digital Vollständig digital

___________________________________________________________________

1. In welchem Ausmaß ist Ihr Zeitmanagement digital?

Überhaupt nicht digital Vollständig digital

___________________________________________________________________

**German version of the QDES**

Fragebogen zur Qualität digitaler Erfahrungen

Die folgenden Aussagen beziehen sich auf Ihre Erfahrungen mit der digitalen Technologie.

Für die Zwecke dieses Fragebogens definieren wir digitale Technologie als **Geräte, Systeme, Dienste, Daten oder Prozesse, die in irgendeiner Weise digitale Informationen nutzen, z. B. Computer, Tablets, Mobiltelefone oder Smart-TVs und andere Geräte**. Diese Technologien können für verschiedene Zwecke wie Kommunikation, Unterhaltung, Arbeit, Bildung und alltägliche Aufgaben verwendet werden.

Lesen Sie jede Aussage und geben Sie an, **in welchem Ausmaß Sie zustimmen**, indem Sie eine Antwort aus den angebotenen Optionen von "stimme gar nicht zu" bis "stimme voll und ganz zu" auswählen.

Bitte antworten Sie so ehrlich wie möglich und denken Sie dabei an Ihre persönlichen Erfahrungen. Es gibt keine richtigen oder falschen Antworten, wir möchten Ihre persönlichen Erfahrungen wissen.

|  | 1. Stimme gar nicht zu | 2. Stimme nicht zu | 3. Weder noch | 4. Stimme zu | 5. Stimme voll und ganz zu |
| --- | --- | --- | --- | --- | --- |
| 1. Die Nutzung digitaler Technologien hilft mir, die Beziehungen zu anderen zu vertiefen | ○ | ○ | ○ | ○ | ○ |
| 2. Die Nutzung digitaler Technologien steigert mein psychisches Wohlbefinden. | ○ | ○ | ○ | ○ | ○ |
| 3. Durch die Nutzung digitaler Technologien bin ich in der Lage, meine Stimmung zu verbessern | ○ | ○ | ○ | ○ | ○ |
| 4. Die Nutzung digitaler Technologien erleichtert die Erledigung von Aufgaben in meinem Leben | ○ | ○ | ○ | ○ | ○ |
| 5. Die Nutzung digitaler Technologien macht mein Leben angenehmer | ○ | ○ | ○ | ○ | ○ |
| 6. Durch die Nutzung digitaler Technologien fühle ich mich Menschen näher | ○ | ○ | ○ | ○ | ○ |
| 7. Die Nutzung digitaler Technologien ermöglicht es mir, mit weniger Aufwand mehr zu erreichen | ○ | ○ | ○ | ○ | ○ |
| 8. Durch die Nutzung digitaler Technologien spare ich Zeit bei der Erledigung von alltäglichen Aufgaben | ○ | ○ | ○ | ○ | ○ |
| 9. Durch die Nutzung digitaler Technologien fühle ich mich von anderen Menschen unterstützt | ○ | ○ | ○ | ○ | ○ |
| 10. Die Nutzung digitaler Technologien hilft mir, mich als Teil des Lebens anderer Menschen zu fühlen | ○ | ○ | ○ | ○ | ○ |
| 11. Durch die Nutzung digitaler Technologien fühle ich mich mit anderen Menschen stärker verbunden | ○ | ○ | ○ | ○ | ○ |
| 12. Die Nutzung digitaler Technologien hilft mir, mich mit anderen zu verbinden | ○ | ○ | ○ | ○ | ○ |
| 13. Die Nutzung digitaler Technologien hilft mir, mich um mein psychisches Wohlbefinden zu kümmern | ○ | ○ | ○ | ○ | ○ |
| 14. Die Nutzung digitaler Technologien verstärkt mein Zugehörigkeitsgefühl zu einer Gruppe | ○ | ○ | ○ | ○ | ○ |
| 15. Die Nutzung digitaler Technologien stärkt meine Verbundenheit zu anderen | ○ | ○ | ○ | ○ | ○ |
| 16. Die Nutzung digitaler Technologien ist zeiteinsparend | ○ | ○ | ○ | ○ | ○ |
| 17. Die Nutzung digitaler Technologien macht mein soziales Leben erfüllter | ○ | ○ | ○ | ○ | ○ |
| 18. Die Nutzung digitaler Technologien hilft mir, mich zu entspannen | ○ | ○ | ○ | ○ | ○ |
| 19. Die Nutzung digitaler Technologien hilft mir, meine Bedürfnisse schneller zu erfüllen | ○ | ○ | ○ | ○ | ○ |
| 20. Durch die Nutzung digitaler Technologien fühle ich mich Menschen wie mir näher | ○ | ○ | ○ | ○ | ○ |
| 21. Durch die Nutzung digitaler Technologien erledige ich Aufgaben schneller | ○ | ○ | ○ | ○ | ○ |
| 22. Durch die Nutzung digitaler Technologien erreiche ich mehr in kürzerer Zeit | ○ | ○ | ○ | ○ | ○ |
| 23. Die Nutzung digitaler Technologien hilft mir, Aufgaben effizient zu erledigen | ○ | ○ | ○ | ○ | ○ |
| 24. Die Nutzung digitaler Technologien erleichtert den Aufbau von tiefen Verbindungen zu anderen | ○ | ○ | ○ | ○ | ○ |
| 25. Durch die Nutzung der digitalen Technologien erlebe ich ein größeres Gemeinschaftsgefühl | ○ | ○ | ○ | ○ | ○ |
| 26. Die Nutzung digitaler Technologien erleichtert mein Leben | ○ | ○ | ○ | ○ | ○ |

**French version of the IDLS**

Échelle d’immersion dans la vie digitale

Les questions suivantes portent sur la mesure dans laquelle vous utilisez la technologie digitale dans votre vie. Pour les besoins de ce questionnaire, nous définissons la technologie digitale comme **des appareils, systèmes, services, données ou processus qui utilisent l’information digitale d’une certaine manière, par exemple des ordinateurs, tablettes, téléphones mobiles ou télévisions connectées, parmi d’autres appareils.** Ces technologies peuvent être utilisées à des fins diverses telles que la communication, le divertissement, le travail, l’éducation et les tâches quotidiennes.

Lisez chacune des questions et répondez aussi honnêtement que possible par rapport à votre expérience personnelle. Veuillez faire glisser le curseur sous chaque question pour indiquer **dans quelle mesure** chaque activité de la vie est **digitale** pour vous. Il n’y a pas de bonnes ou de mauvaises réponses, nous voulons connaître votre expérience personnelle.

1. Dans quelle mesure vos relations sociales sont-elles menées de manière digitale?

Pas du tout digital Complètement digital

___________________________________________________________________

1. Dans quelle mesure votre communication avec la famille est-elle digitale?

Pas du tout digital Complètement digital

___________________________________________________________________

1. Dans quelle mesure les activités que vous faites dans votre temps libre sont-elles digitales?

Pas du tout digital Complètement digital

___________________________________________________________________

1. Dans quelle mesure votre communication avec les amis est-elle digitale?

Pas du tout digital Complètement digital

___________________________________________________________________

1. Dans quelle mesure la gestion de votre temps est-elle digitale?

Pas du tout digital Complètement digital

___________________________________________________________________

**French version of the QDES**

Échelle de la qualité de l’expérience digitale

Les questions suivantes portent sur votre expérience vécue avec la technologie digitale.

Pour les besoins de ce questionnaire, nous définissons la technologie digitale comme **des appareils, systèmes, services, données ou processus qui utilisent l’information digitale d’une certaine manière, par exemple des ordinateurs, tablettes, téléphones mobiles ou télévisions connectées, parmi d’autres appareils.** Ces technologies peuvent être utilisées à des fins diverses telles que la communication, le divertissement, le travail, l’éducation et les tâches quotidiennes.

Lisez chaque affirmation et indiquez **dans quelle mesure vous êtes d’accord** en sélectionnant **une réponse** parmi les options proposées allant de « fortement en désaccord » à « fortement d'accord ».

Veuillez répondre aussi honnêtement que possible en pensant à votre expérience personnelle. Il n'y a pas de bonnes ou de mauvaises réponses, nous voulons connaître votre expérience personnelle.

|  | 1. Fortement en désaccord | 2. En désaccord | 3. Ni d’accord ni en désaccord | 4. D’accord | 5. Fortement d’accord |
| --- | --- | --- | --- | --- | --- |
| 1. Utiliser la technologie digitale m’aide à approfondir les relations avec les autres | ○ | ○ | ○ | ○ | ○ |
| 2. Utiliser la technologie digitale améliore mon bien-être psychologique | ○ | ○ | ○ | ○ | ○ |
| 3. Je suis capable d’améliorer mon humeur en utilisant la technologie digitale | ○ | ○ | ○ | ○ | ○ |
| 4. Utiliser la technologie digitale facilite l’accomplissement des tâches dans ma vie | ○ | ○ | ○ | ○ | ○ |
| 5. Utiliser la technologie digitale rend ma vie agréable | ○ | ○ | ○ | ○ | ○ |
| 6. Utiliser la technologie digitale fait que je me sens plus proche des gens | ○ | ○ | ○ | ○ | ○ |
| 7. Utiliser la technologie digitale me permet d’en faire plus avec moins d’efforts | ○ | ○ | ○ | ○ | ○ |
| 8. En utilisant la technologie digitale, je gagne du temps sur les tâches quotidiennes | ○ | ○ | ○ | ○ | ○ |
| 9. Utiliser la technologie digitale fait que je me sens soutenu-e par d’autres personnes | ○ | ○ | ○ | ○ | ○ |
| 10. Utiliser la technologie digitale m’aide à sentir que je fais partie de la vie des autres | ○ | ○ | ○ | ○ | ○ |
| 11. Utiliser la technologie digitale fait que je me sens plus connecté-e aux autres personnes | ○ | ○ | ○ | ○ | ○ |
| 12. Utiliser la technologie digitale m'aide à créer des liens avec les autres | ○ | ○ | ○ | ○ | ○ |
| 13. Utiliser la technologie digitale m’aide à prendre soin de mon bien-être psychologique | ○ | ○ | ○ | ○ | ○ |
| 14. Utiliser la technologie digitale augmente mon sentiment d’appartenance à un groupe | ○ | ○ | ○ | ○ | ○ |
| 15. Utiliser la technologie digitale renforce mon attachement aux autres | ○ | ○ | ○ | ○ | ○ |
| 16. Utiliser la technologie digitale me fait gagner du temps | ○ | ○ | ○ | ○ | ○ |
| 17. Utiliser la technologie digitale rend ma vie sociale plus épanouissante | ○ | ○ | ○ | ○ | ○ |
| 18. Utiliser la technologie digitale m’aide à me détendre | ○ | ○ | ○ | ○ | ○ |
| 19. Je peux répondre à mes besoins plus rapidement en utilisant la technologie digitale | ○ | ○ | ○ | ○ | ○ |
| 20. Je me sens plus proche des gens comme moi parce que j'utilise la technologie digitale | ○ | ○ | ○ | ○ | ○ |
| 21. J’accomplis des tâches plus rapidement quand j’utilise la technologie digitale | ○ | ○ | ○ | ○ | ○ |
| 22. Je réalise plus de choses en moins de temps quand j’utilise la technologie digitale | ○ | ○ | ○ | ○ | ○ |
| 23. Utiliser la technologie digitale m’aide à accomplir des tâches efficacement | ○ | ○ | ○ | ○ | ○ |
| 24. Je trouve facile d'établir des liens profonds avec les autres lorsque j'utilise la technologie digitale | ○ | ○ | ○ | ○ | ○ |
| 25. Je fais l’expérience d’un plus grand sentiment d’appartenance à une communauté grâce à l'utilisation de la technologie digitale | ○ | ○ | ○ | ○ | ○ |
| 26. Utiliser la technologie digitale rend ma vie plus facile | ○ | ○ | ○ | ○ | ○ |

**Spanish version of the IDLS**

Escala de Inmersión en la Vida Digital

Las siguientes preguntas se refieren a la medida en que usted utiliza tecnología digital en su vida. Para los fines de este cuestionario, tecnología digital se define como **dispositivos, sistemas, servicios, datos o procesos que utilizan información digital de alguna manera, por ejemplo, ordenadores, tabletas, teléfonos móviles o televisores inteligentes, entre otros dispositivos**. Estas tecnologías pueden utilizarse para diversos fines, tales como comunicación, entretenimiento, trabajo, educación y tareas cotidianas.

Lea cada una de las preguntas y responda lo más honestamente que pueda, teniendo en cuenta su experiencia personal. Por favor, deslice y sitúe el cursor debajo de cada pregunta para indicar **el nivel de digitalización** que tiene para usted cada una de las actividades de su vida. No hay respuestas buenas ni malas, queremos conocer su experiencia personal.

1. ¿En qué medida sus relaciones sociales se llevan a cabo de forma digital?

Nada digital Completamente digital

___________________________________________________________________

1. ¿En qué medida es digital su comunicación con la familia?

Nada digital Completamente digital

___________________________________________________________________

1. ¿En qué medida son digitales las actividades que hace en su tiempo libre?

Nada digital Completamente digital

___________________________________________________________________

1. ¿En qué medida es digital la comunicación con sus amistades?

Nada digital Completamente digital

___________________________________________________________________

1. ¿En qué medida es digital la gestión de su tiempo?

Nada digital Completamente digital

___________________________________________________________________

**Spanish version of the QDES**

Escala de Calidad de la Experiencia Digital

Las siguientes preguntas se refieren a la experiencia directa que usted ha tenido con la tecnología digital.

Para los fines de este cuestionario, tecnología digital se define como **dispositivos, sistemas, servicios, datos o procesos que utilizan información digital de alguna manera, por ejemplo, ordenadores, tabletas, teléfonos móviles o televisores inteligentes, entre otros dispositivos**. Estas tecnologías pueden utilizarse para diversos fines, tales como comunicación, entretenimiento, trabajo, educación y tareas cotidianas.

Lea cada afirmación e indique **en qué medida está de acuerdo con ella**; para ello seleccione **una respuesta** entre las opciones proporcionadas, que van desde "muy en desacuerdo" hasta "muy de acuerdo".

Por favor, responda lo más honestamente que pueda, teniendo en cuenta su propia experiencia. No hay respuestas buenas ni malas, sólo se trata de conocer su experiencia personal.

|  | 1. Muy en desacuerdo | 2. En desacuerdo | 3. Ni de acuerdo, ni en desacuerdo | 4. De acuerdo | 5. Muy de acuerdo |
| --- | --- | --- | --- | --- | --- |
| 1. Usar tecnología digital me ayuda a profundizar mis relaciones con los demás | ○ | ○ | ○ | ○ | ○ |
| 2. Usar tecnología digital mejora mi bienestar mental | ○ | ○ | ○ | ○ | ○ |
| 3. Puedo mejorar mi estado de ánimo usando tecnología digital | ○ | ○ | ○ | ○ | ○ |
| 4. Usar tecnología digital me facilita la realización de tareas en mi vida | ○ | ○ | ○ | ○ | ○ |
| 5. Usar tecnología digital hace que mi vida sea placentera | ○ | ○ | ○ | ○ | ○ |
| 6. Usar tecnología digital me hace sentir más cercano a las demás personas | ○ | ○ | ○ | ○ | ○ |
| 7. Usar la tecnología digital me permite hacer más con menos esfuerzo | ○ | ○ | ○ | ○ | ○ |
| 8. Al utilizar tecnología digital ahorro tiempo en las tareas cotidianas | ○ | ○ | ○ | ○ | ○ |
| 9. Usar tecnología digital me hace sentir apoyado por otras personas | ○ | ○ | ○ | ○ | ○ |
| 10. Usar tecnología digital me ayuda a sentirme parte de la vida de los demás | ○ | ○ | ○ | ○ | ○ |
| 11. Usar tecnología digital me hace sentir más conectado con otras personas | ○ | ○ | ○ | ○ | ○ |
| 12. Usar tecnología digital me ayuda crear vínculos con los demás | ○ | ○ | ○ | ○ | ○ |
| 13. Usar tecnología digital me ayuda a cuidar de mi bienestar mental | ○ | ○ | ○ | ○ | ○ |
| 14. El uso de tecnología digital incrementa mi sentido de pertenencia a un grupo | ○ | ○ | ○ | ○ | ○ |
| 15. Usar tecnología digital fortalece mis vínculos con otras personas | ○ | ○ | ○ | ○ | ○ |
| 16. Usar tecnología digital me ahorra tiempo | ○ | ○ | ○ | ○ | ○ |
| 17. Usar tecnología digital hace que mi vida social sea más satisfactoria | ○ | ○ | ○ | ○ | ○ |
| 18. Usar tecnología digital me ayuda a relajarme | ○ | ○ | ○ | ○ | ○ |
| 19. Puedo satisfacer mis necesidades más rápidamente utilizando tecnología digital | ○ | ○ | ○ | ○ | ○ |
| 20. Siento más cercanía con personas como yo porque utilizo tecnología digital | ○ | ○ | ○ | ○ | ○ |
| 21. Completo las tareas más rápidamente cuando utilizo tecnología digital | ○ | ○ | ○ | ○ | ○ |
| 22. Logro más en menos tiempo cuando uso tecnología digital | ○ | ○ | ○ | ○ | ○ |
| 23. Usar tecnología digital me ayuda a completar tareas de manera eficiente | ○ | ○ | ○ | ○ | ○ |
| 24. Me resulta fácil crear conexiones profundas con otras personas cuando uso tecnología digital | ○ | ○ | ○ | ○ | ○ |
| 25. Experimento un mayor sentimiento de comunidad gracias al uso de tecnología digital | ○ | ○ | ○ | ○ | ○ |
| 26. Usar tecnología digital hace que mi vida sea más fácil | ○ | ○ | ○ | ○ | ○ |

**Polish version of the IDLS**

Skala zanurzenia w cyfrowym świecie

Poniższe pytania dotyczą stopnia, w jakim korzystasz z technologii cyfrowej w swoim życiu. Na potrzeby tego kwestionariusza definiujemy technologię cyfrową jako **urządzenia, systemy, usługi, dane lub procesy, które w jakikolwiek sposób wykorzystują informacje cyfrowe, na przykład komputery, tablety, telefony komórkowe i inteligentne telewizory oraz inne tego typu urządzenia**. Technologie te mogą być wykorzystywane do różnych celów, takich jak komunikacja, rozrywka, praca, edukacja i codzienne zadania.

Przeczytaj poniższe pytania i odpowiedz na nie tak szczerze, jak tylko potrafisz, myśląc o swoich osobistych doświadczeniach. Proszę przeciągnij suwak pod każdym pytaniem, aby wskazać **jak bardzo ucyfryzowana** jest dla Ciebie każda czynność życiowa. Nie ma dobrych ani złych odpowiedzi, chcemy poznać Twoje osobiste doświadczenia.

1. W jakim stopniu Twoje relacje społeczne są prowadzone przy użyciu technologii cyfrowej?

Wcale nie cyfrowo Całkowicie cyfrowo

___________________________________________________________________

1. W jakim stopniu Twoja komunikacja z rodziną odbywa się cyfrowo?

Wcale nie cyfrowo Całkowicie cyfrowo

___________________________________________________________________

1. W jakim stopniu czynności, które wykonujesz w czasie wolnym są cyfrowe?

Wcale nie cyfrowo Całkowicie cyfrowo

___________________________________________________________________

1. W jakim stopniu Twoja komunikacja ze znajomymi odbywa się cyfrowo?

Wcale nie cyfrowo Całkowicie cyfrowo

___________________________________________________________________

1. W jakim stopniu zarządzanie Twoim czasem odbywa się cyfrowo?

Wcale nie cyfrowo Całkowicie cyfrowo

___________________________________________________________________

**Polish version of the QDES**

Skala jakości doświadczenia cyfrowego

Poniższe pytania dotyczą Twoich osobistych doświadczeń z technologią cyfrową.

Na potrzeby tego kwestionariusza definiujemy technologię cyfrową jako **urządzenia, systemy, usługi, dane lub procesy, które w jakikolwiek sposób wykorzystują informacje cyfrowe, na przykład komputery, tablety, telefony komórkowe i inteligentne telewizory oraz inne tego typu urządzenia**. Technologie te mogą być wykorzystywane do różnych celów, takich jak komunikacja, rozrywka, praca, edukacja i codzienne zadania.

Przeczytaj każde poniższe stwierdzenie i zaznacz **w jakim stopniu się z nim zgadzasz** wybierając **jedną odpowiedź** z podanych opcji od "zdecydowanie się nie zgadzam" do "zdecydowanie się zgadzam".

Proszę odpowiedz tak szczerze, jak tylko potrafisz, myśląc o swoich osobistych doświadczeniach. Nie ma dobrych ani złych odpowiedzi, chcemy poznać Twoje osobiste doświadczenia.

|  | 1. Zdecydowanie się nie zgadzam | 2. Nie zgadzam się | 3. Ani się zgadzam, ani się nie zgadzam | 4. Zgadzam się | 5.Zdecydowanie się zgadzam |
| --- | --- | --- | --- | --- | --- |
| 1. Korzystanie z technologii cyfrowej pomaga mi pogłębiać relacje z innymi | ○ | ○ | ○ | ○ | ○ |
| 2. Korzystanie z technologii cyfrowej poprawia moje samopoczucie psychiczne | ○ | ○ | ○ | ○ | ○ |
| 3. Jestem w stanie poprawić sobie nastrój za pomocą technologii cyfrowej | ○ | ○ | ○ | ○ | ○ |
| 4. Korzystanie z technologii cyfrowej ułatwia realizację zadań w moim życiu | ○ | ○ | ○ | ○ | ○ |
| 5. Korzystanie z technologii cyfrowej sprawia, że moje życie jest przyjemne | ○ | ○ | ○ | ○ | ○ |
| 6. Korzystanie z technologii cyfrowej sprawia, że czuję się bliżej ludzi | ○ | ○ | ○ | ○ | ○ |
| 7. Korzystanie z technologii cyfrowej pozwala mi robić więcej przy mniejszym wysiłku | ○ | ○ | ○ | ○ | ○ |
| 8. Korzystając z technologii cyfrowej oszczędzam czas na codziennych zadaniach | ○ | ○ | ○ | ○ | ○ |
| 9. Korzystanie z technologii cyfrowej sprawia, że czuję wsparcie od innych ludzi | ○ | ○ | ○ | ○ | ○ |
| 10. Korzystanie z technologii cyfrowej pomaga mi czuć się częścią życia innych ludzi | ○ | ○ | ○ | ○ | ○ |
| 11. Korzystanie z technologii cyfrowej sprawia, że czuję się bardziej związany/a z innymi ludźmi | ○ | ○ | ○ | ○ | ○ |
| 12. Korzystanie z technologii cyfrowej pomaga mi nawiązywać więzi z innymi | ○ | ○ | ○ | ○ | ○ |
| 13. Korzystanie z technologii cyfrowej pomaga mi dbać o dobre samopoczucie psychiczne | ○ | ○ | ○ | ○ | ○ |
| 14. Korzystanie z technologii cyfrowej zwiększa moje poczucie przynależności do grupy | ○ | ○ | ○ | ○ | ○ |
| 15. Korzystanie z technologii cyfrowej wzmacnia moje przywiązanie do innych | ○ | ○ | ○ | ○ | ○ |
| 16. Korzystanie z technologii cyfrowej oszczędza mój czas | ○ | ○ | ○ | ○ | ○ |
| 17. Korzystanie z technologii cyfrowej sprawia, że moje życie towarzyskie jest bardziej satysfakcjonujące | ○ | ○ | ○ | ○ | ○ |
| 18. Korzystanie z technologii cyfrowej pomaga mi się zrelaksować | ○ | ○ | ○ | ○ | ○ |
| 19. Dzięki technologii cyfrowej mogę szybciej zaspokajać swoje potrzeby | ○ | ○ | ○ | ○ | ○ |
| 20. Czuję się bliżej ludzi takich, jak ja, ponieważ korzystam z technologii cyfrowej | ○ | ○ | ○ | ○ | ○ |
| 21. Wykonuję zadania szybciej, korzystając z technologii cyfrowej | ○ | ○ | ○ | ○ | ○ |
| 22. Osiągam więcej w krótszym czasie, gdy korzystam z technologii cyfrowej | ○ | ○ | ○ | ○ | ○ |
| 23. Korzystanie z technologii cyfrowej pomaga mi efektywnie wykonywać zadania | ○ | ○ | ○ | ○ | ○ |
| 24. Łatwiej mi kształtować głębokie relacje z innymi, korzystając z technologii cyfrowej | ○ | ○ | ○ | ○ | ○ |
| 25. Doświadczam większego poczucia wspólnoty, dzięki korzystaniu z technologii cyfrowej | ○ | ○ | ○ | ○ | ○ |
| 26. Korzystanie z technologii cyfrowej ułatwia mi życie | ○ | ○ | ○ | ○ | ○ |

**Czech version of the IDLS**

Škála zapojení do digitálního života

Následující otázky se týkají toho, do jaké míry ve svém životě používáte digitální technologie. Pro účely tohoto dotazníku definujeme digitální technologie jako **zařízení, systémy, služby, data nebo procesy, které nějakým způsobem využívají digitální informace, například počítače, tablety, mobilní telefony nebo chytré televize a další zařízení**. Tyto technologie mohou být používány k různým účelům, jako je komunikace, zábava, práce, vzdělávání a každodenní úkoly.

Přečtěte si každou z otázek a odpovězte co nejupřímněji na základě svých osobních zkušeností. Přetažením posuvníku pod každou otázkou prosím označte, **nakolik digitální** jsou pro Vás jednotlivé činnosti. Žádná odpověď není dobrá ani špatná, zajímá nás Vaše osobní zkušenost.

1. Do jaké míry se Vaše mezilidské vztahy odehrávají digitálně?

Vůbec ne digitálně Zcela digitálně

___________________________________________________________________

1. Do jaké míry je komunikace s Vaší rodinou digitální?

Vůbec ne digitální Zcela digitální

___________________________________________________________________

1. Do jaké míry jsou Vaše volnočasové aktivity digitální?

Vůbec ne digitální Zcela digitální

___________________________________________________________________

1. Do jaké míry je komunikace s Vašimi přáteli digitální?

Vůbec ne digitální Zcela digitální

___________________________________________________________________

1. Do jaké míry si plánujete čas digitálně?

Vůbec ne digitálně Zcela digitálně

___________________________________________________________________

**Czech version of the QDES**

Škála kvality digitální zkušenosti

Následující otázky se týkají Vašich zkušeností s digitálními technologiemi.

Pro účely tohoto dotazníku definujeme digitální technologie jako **zařízení, systémy, služby, data nebo procesy, které nějakým způsobem využívají digitální informace, například počítače, tablety, mobilní telefony nebo chytré televize a další zařízení**. Tyto technologie mohou být používány k různým účelům, jako je komunikace, zábava, práce, vzdělávání a každodenní úkoly.

Přečtěte si jednotlivá tvrzení a u každého označte, **do jaké míry s ním souhlasíte** výběrem **jedné odpovědi** z nabízených možností od „rozhodně nesouhlasím“ po „rozhodně souhlasím“.

Odpovězte prosím co nejupřímněji na základě svých osobních zkušeností. Žádná odpověď není dobrá ani špatná, zajímá nás Vaše osobní zkušenost.

|  | 1.  Rozhodně nesouhlasím | 2.  Nesouhlasím | 3.  Ani souhlasím, ani nesouhlasím | 4. Souhlasím | 5.  Rozhodně souhlasím |
| --- | --- | --- | --- | --- | --- |
| 1. Používání digitálních technologií mi pomáhá prohlubovat vztahy s ostatními. | ○ | ○ | ○ | ○ | ○ |
| 2. Používání digitálních technologií zlepšuje mou duševní pohodu. | ○ | ○ | ○ | ○ | ○ |
| 3. Pomocí digitálních technologií si dokážu zlepšit náladu. | ○ | ○ | ○ | ○ | ○ |
| 4. Používání digitálních technologií mi usnadňuje plnění každodenních úkolů. | ○ | ○ | ○ | ○ | ○ |
| 5. Používání digitálních technologií mi zpříjemňuje život. | ○ | ○ | ○ | ○ | ○ |
| 6. Používání digitálních technologií mi dává pocit blízkosti s druhými. | ○ | ○ | ○ | ○ | ○ |
| 7. Používání digitálních technologií mi umožňuje dělat více s menším úsilím. | ○ | ○ | ○ | ○ | ○ |
| 8. Používáním digitálních technologií šetřím čas při plnění každodenních úkolů. | ○ | ○ | ○ | ○ | ○ |
| 9. Používání digitálních technologií mi dodává pocit podpory od druhých. | ○ | ○ | ○ | ○ | ○ |
| 10. Používání digitálních technologií mi pomáhá cítit se součástí života druhých. | ○ | ○ | ○ | ○ | ○ |
| 11. Používání digitálních technologií mi dává pocit většího propojení s druhými. | ○ | ○ | ○ | ○ | ○ |
| 12. Používání digitálních technologií mi pomáhá sbližovat se s druhými. | ○ | ○ | ○ | ○ | ○ |
| 13. Používání digitálních technologií mi pomáhá pečovat o svou duševní pohodu. | ○ | ○ | ○ | ○ | ○ |
| 14. Používání digitálních technologií prohlubuje můj pocit sounáležitosti se skupinou. | ○ | ○ | ○ | ○ | ○ |
| 15. Používání digitálních technologií posiluje mé vazby k druhým. | ○ | ○ | ○ | ○ | ○ |
| 16. Používáním digitálních technologií šetřím čas. | ○ | ○ | ○ | ○ | ○ |
| 17. Díky používání digitálních technologií je můj společenský život více naplňující. | ○ | ○ | ○ | ○ | ○ |
| 18. Používání digitálních technologií mi pomáhá uvolnit se. | ○ | ○ | ○ | ○ | ○ |
| 19. Díky používání digitálních technologií dokážu rychleji uspokojovat své potřeby. | ○ | ○ | ○ | ○ | ○ |
| 20. Díky používání digitálních technologií se cítím být blíže lidem, kteří jsou mi podobní. | ○ | ○ | ○ | ○ | ○ |
| 21. Při používání digitálních technologií plním úkoly rychleji. | ○ | ○ | ○ | ○ | ○ |
| 22. Když používám digitální technologie, stihnu toho více za kratší dobu. | ○ | ○ | ○ | ○ | ○ |
| 23. Díky používání digitálních technologií plním úkoly efektivněji. | ○ | ○ | ○ | ○ | ○ |
| 24. Při používání digitálních technologií je pro mě snadné navázat hluboké vztahy s druhými. | ○ | ○ | ○ | ○ | ○ |
| 25. Díky používání digitálních technologií zažívám větší pocit sounáležitosti. | ○ | ○ | ○ | ○ | ○ |
| 26. Používání digitálních technologií mi usnadňuje život. | ○ | ○ | ○ | ○ | ○ |
